# Full Discretisations for Nonlinear Evolutionary Inequalities Based on Stiffly Accurate Runge–Kutta and *hp*-Finite Element Methods

**DOI:** 10.1007/s10208-013-9179-3

**Published:** 2013-11-13

**Authors:** J. Gwinner, M. Thalhammer

**Affiliations:** 1 Institut für Mathematik und Rechneranwendung, Universität der Bundeswehr München, Werner-Heisenberg-Weg 39, 85577 Neubiberg, Germany; 2 Institut für Mathematik, Leopold–Franzens Universität Innsbruck, Technikerstrasse 13/7, 6020 Innsbruck, Austria

**Keywords:** Nonlinear evolutionary inequalities, Nonlinear differential inclusions, Monotone operators, Stiffly accurate Runge–Kutta methods, Nonconforming Galerkin methods, *hp*-finite element approximations, Stability, Convergence, 35K61, 35K86, 47J05, 47J20, 47J22, 47H05, 65M12

## Abstract

The convergence of full discretisations by implicit Runge–Kutta and nonconforming Galerkin methods applied to nonlinear evolutionary inequalities is studied. The scope of applications includes differential inclusions governed by a nonlinear operator that is monotone and fulfills a certain growth condition. A basic assumption on the considered class of stiffly accurate Runge–Kutta time discretisations is a stability criterion which is in particular satisfied by the Radau IIA and Lobatto IIIC methods. In order to allow nonconforming *hp*-finite element approximations of unilateral constraints, set convergence of convex subsets in the sense of Glowinski–Mosco–Stummel is utilised. An appropriate formulation of the fully discrete variational inequality is deduced on the basis of a characteristic example of use, a Signorini-type initial-boundary value problem. Under hypotheses close to the existence theory of nonlinear first-order evolutionary equations and inequalities involving a monotone main part, a convergence result for the piecewise constant in time interpolant is established.

## Introduction

### Scope of Applications

Nowadays, transient heat transfer between different materials under nonlinear unilateral/bilateral transmission conditions [13] and time-dependent contact between deformable bodies with possibly nonlinear material behaviour [14, 35] receive great attention because of relevant applications in engineering. Time-dependent free boundary problems of a similar character occur in elastoplasticity [23], often connected with viscosity, damage, fatigue and other effects [36].

### Time and Space Discretisations

Discretisations for related classes of problems that are based on implicit time integration methods and a Galerkin approach in space arouse the interest of many researchers. Numerous contributions confirm that implicit Runge–Kutta and linear multistep methods are favourable for the time discretisation of dynamic processes described by nonlinear evolution equations; as a small selection of literature we refer to [7, 15, 22, 37] and the references given therein. In particular, due to the stiff nature of the problems, implicit methods show superior stability properties compared to explicit methods. The behaviour of piecewise linear finite element approximations has been studied among others in [18, 24–26] for the restricted static and quasistatic regime of linear material behaviour in elasticity and plasticity. The convergence of the more general *h*-version finite element method (FEM) with respect to the mesh size *h*, where the order *p* of the finite elements is low and fixed to *p*=2 or *p*=3, respectively, is established in [17, 19] for static unilateral/non-smooth contact problems. Contrary, the *hp*-version finite element method achieves convergence not only by refining the mesh size *h*, but also by increasing the polynomial order *p* of the finite elements. By the pioneering work of Babuška and co-workers, the exponentially fast convergence of the *hp*-finite element method is well-known for linear elliptic problems. More recently, the superior convergence properties of the *p*- and *hp*- compared to the standard *h*-finite element method have been proven for certain nonlinear elliptic problems and demonstrated in numerical experiments. In [1] the nonlinear Laplace equation is treated, and in [27] a domain obstacle problem with a general nonlinear monotone elliptic operator of quadratic growth is studied; for the investigation of unilateral contact problems within the range of linear elasticity without and with Tresca friction and related elliptic variational inequalities, see [11, 12, 16, 21, 28].

### Objective

In the present work, our concern is to combine a class of implicit Runge–Kutta methods for time discretisation and the *hp*-finite element method for space discretisation to arrive at full discretisations of nonlinear first-order in time evolutionary variational inequalities. Our analysis covers problems involving a main part that is monotone and satisfies a certain, not necessarily quadratic, growth condition. A basic assumption on the considered class of stiffly accurate Runge–Kutta time discretisations is a stability criterion which implies algebraic stability and is in particular fulfilled by the widely used Radau IIA method, including as special case the implicit Euler method, and by the Lobatto IIIC methods. In order to allow *hp*-finite element approximations that lead to nonconforming approximations of the given unilateral constraints, we utilise set convergence of convex subsets in the sense of Glowinski–Mosco–Stummel [17, 29, 38]. On the basis of a relatively simple model application, a Signorini-type initial-boundary value problem, we deduce an appropriate formulation of the fully discrete variational inequality. Under hypotheses close to the existence theory of nonlinear evolutionary inequalities, dispensing with unnatural regularity requirements on the exact solution to the problem, we are able to establish a convergence result for the piecewise constant in time interpolant.

The present work generalises [8], where full discretisations based on the implicit Euler method and low-order finite element approximations applied to nonlinear evolutionary inequalities are studied. Moreover, it extends the analysis of stiffly accurate Runge–Kutta time discretisations for nonlinear evolution equations given in [15] to nonlinear evolutionary inequalities.

To our knowledge, this work is the first contribution, where a class of implicit Runge–Kutta methods is investigated for nonlinear evolutionary inequalities. For this reason, we justify in more detail the employed novel formulation of the fully discrete variational inequality and deduce the needed auxiliary results for stiffly accurate Runge–Kutta and *hp*-finite element discretisations. However, to keep the work at a reasonable length, in the convergence proof we only indicate the standard arguments used and refer to the literature for further details.

### Outline

The work has the following structure. In Sects. 2 and 3 we state a characteristic model problem, a Signorini-type initial-boundary value problem, deduce its formulation as variational inequality, and specify the considered class of time and space discretisations. In Sects. 4 and 5, we introduce a general analytical framework of nonlinear evolutionary variational inequalities and its fully discrete counterpart; in particular, we deduce a result on the existence and uniqueness of the fully discrete solution as well as a priori bounds for the discrete approximation values. A convergence result for the piecewise constant in time interpolant related to the relaxed formulation of the evolutionary variational inequality is finally established in Sect. 6.

### Notations

To conclude the introduction we collect auxiliary notations that are used throughout. For further details on the considered function spaces, we refer to [9].

We employ standard vector and matrix notations such as $x = (x_{1},\dots ,x_{d}) \in \mathbb {R}^{d}$ and $\mathfrak {A}= (a_{ij})_{1 \leq i,j \leq s} \in \mathbb {R}^{s \times s}$. The Euclidian inner product and the associated norm are given by *x*⋅*y*=(*x*|*y*)=*x*_1_ *y*_1_+⋯+*x*_*d*_ *y*_*d*_ and $\|x\| = \sqrt{(x|x)}$ for $x,y \in \mathbb {R}^{d}$. The partial derivative with respect to *x*_*i*_ is denoted by $\partial _{x_{i}}$, and, as usual, we employ the abbreviations ∇, div, Δ for the Nabla operator, divergence and Laplacian.

Throughout, we consider a finite time interval $I = (0,T) \subset \mathbb {R}$, where *T*>0. We suppose that the spatial domain $\varOmega \subset \mathbb {R}^{d}$ with *d*=2 or *d*=3 is bounded and Lipschitz; to simplify matters, in connection with the finite element approximation we consider a polyhedral domain. The Dirichlet boundary condition and the unilateral constraint are prescribed on the sets *Γ*_*D*_ and *Γ*_*S*_, respectively, where we suppose $\partial \varOmega = \varGamma = \overline {\varGamma }_{D}\cup \overline {\varGamma }_{S}$ and *Γ*_*D*_∩*Γ*_*S*_=∅. We require the Dirichlet boundary to be of positive measure, i.e. $\operatorname{meas} \varGamma _{D}> 0$, and, for simplicity of the exposition, we do not prescribe Neumann boundary conditions on a boundary part, although this would pose no additional difficulty in the analysis.

We suppose that the exponent, which arises in the Signorini-type initial-boundary value problem or the hypotheses on the functional defining the variational inequality, respectively, satisfies *r*∈[2,∞); the associated exponent is denoted by $r^{*}= \frac{r}{r-1}$ and evidently satisfies *r* ^∗^∈(1,2].

The space of continuous functions  and the Lebesgue and Sobolev spaces such as *L* ^2^(*Ω*), *W* ^1,*r*^(*Ω*) and $W^{-1,r^{*}}(\varOmega )$ are equipped with the standard norms.

We recall that a Gelfand triple *X*⊂*H*⊂*X* ^∗^ is formed by a real and separable Hilbert space (*H*,(⋅|⋅)_*H*_,∥⋅∥_*H*_) and a real, separable and reflexive Banach space (*X*,∥⋅∥_*X*_) such that *X* is dense and continuously embedded in *H*. As standard, the dual space $(X^{*},\|\cdot\|_{X^{*}})$ is equipped with the norm $\|x^{*}\|_{X^{*}} = \sup\{|x^{*}(x)|: x \in X, \|x\|_{X} = 1\}$, and for *x*∈*X* and *x* ^∗^∈*X* ^∗^ the duality pairing is given by $\langle x^{*}\vert x\rangle _{X^{*}\times X} = x^{*}(x)$, which extends the inner product (⋅|⋅)_*H*_.

For exponents *q*∈[1,∞) and a Banach space *Z* the space *L* ^*q*^(*I*,*Z*) of Bochner integrable abstract functions *z*:*I*→*Z* is endowed with the norm

$$ \|z\|_{L^q(I,Z)}^q = \int_{0}^{\mathrm{T}} \bigl\| z(t)\bigr\| _{Z}^q \,\mathrm {d}t, $$

and the space  of *Z*-valued continuous functions on $\overline {I}$ is equipped with the topology of uniform convergence. For elements *z*∈*L* ^*q*^(*I*,*Z*) and $z^{*} \in(L^{q}(I,Z))^{*} = L^{q^{*}}(I,Z^{*})$ with $q^{*} = \frac{q}{q-1}$ the duality pairing is defined by

$$ \bigl\langle z^{*} \big\vert z \bigr\rangle _{L^{q^{*}}(I,Z^{*}) \times L^q(I,Z)} = \int_{0}^{\mathrm{T}} \bigl\langle z^{*}(t) \big\vert z(t) \bigr\rangle _{Z^{*}\times Z} \,\mathrm {d}t. $$

As the distinction is clear from the context, we use the same letters for instance for elements in a Banach space *Z* and functions in *L* ^*q*^(*I*,*Z*), respectively.

For a Hilbert space (*Z*,(⋅|⋅)_*Z*_,∥⋅∥_*Z*_) the product space $(Z^{s},(\cdot\vert \cdot)_{Z^{s}},\|\cdot\|_{Z^{s}} )$ is endowed with inner product and corresponding norm given by

$$ (z\vert \widetilde{z})_{Z^s} = \sum _{i = 1}^{s} (z_i\vert \widetilde{z}_i)_{Z}, \qquad \|z\|_{Z^s}^2 = \sum_{i = 1}^{s} \|z_i\|_{Z}^2, $$

for *z*=(*z*_1_,…,*z*_*s*_)∈*Z* ^*s*^ and $\widetilde{z} = (\widetilde {z}_{1}, \dots, \widetilde{z}_{s}) \in Z^{s}$. Besides, for product spaces the duality pairing is defined componentwise; for instance, we set

$$ \bigl\langle z^{*} \big\vert z \bigr\rangle _{(L^{q^{*}}(I,Z^{*}))^s \times (L^q(I,Z))^s} = \sum _{i = 1}^{s} \bigl\langle z_i^{*} \big\vert z_i \bigr\rangle _{L^{q^{*}}(I,Z^{*}) \times L^q(I,Z)} $$

for $z^{*} = (z_{1}^{*},\dots,z_{s}^{*})^{\mathrm{T}} \in(L^{q^{*}}(I,Z^{*}))^{s}$ and *z*=(*z*_1_,…,*z*_*s*_)^T^∈(*L* ^*q*^(*I*,*Z*))^*s*^.

Finally, we denote by *C*>0 a generic constant, possibly with different values at different occasions.

## A Signorini-Type Initial-Boundary Value Problem and Its Formulation as Evolutionary Variational Inequality

In this section, we introduce a simple though characteristic model problem, which shall serve as an illustration of the general framework of nonlinear evolutionary inequalities. We first state the nonlinear parabolic initial-boundary value problem involving the *r*-Laplacian and a free boundary condition of Signorini type and then indicate its reformulation as evolutionary variational inequality. Moreover, we specify the underlying function spaces and the basic properties of the governing operators. We recall that *r*∈[2,∞) and $r^{*}= \frac{r}{r-1}$.

### A Signorini-Type Initial-Boundary Value Problem

We consider the following initial-boundary value problem for a real-valued function $u: \overline {\varOmega }\times \overline {I}\to \mathbb {R}$:

2.1 $$ \begin{gathered} \partial _{t} u - \operatorname{div} \bigl( \|\nabla u\|^{r-2} \nabla u \bigr) = f\quad\text{in } \varOmega \times I, \\ u = g\quad\text{on } \varGamma _{D}\times I, \\ u \geq g,\qquad\|\nabla u\|^{r-2}\partial _{ \mathfrak {n}} u \geq0,\qquad (u-g) \|\nabla u\|^{r-2}\partial _{ \mathfrak {n}} u = 0\quad \text{on } \varGamma _{S}\times I, \\ u = u_0 \quad\text{in } \varOmega \times\{0\}, \end{gathered} $$

involving the given right-hand side $f: \varOmega \times I \to \mathbb {R}$, the (time-independent) function $g: \partial \varOmega \to \mathbb {R}$ defining the boundary condition, as well as the given initial condition *u*_0_.

### Formulation as Evolutionary Variational Inequality

In regard to the fully discrete analogue derived in Sect. 3, we indicate the reformulation of the initial-boundary value problem (2.1) as evolutionary variational inequality. We meanwhile assume the solution $u: \overline {\varOmega }\times \overline {I}\to \mathbb {R}$ to the Signorini-type initial-boundary value problem to be sufficiently regular; the regularity requirements on *u* will be specified below. Moreover, we denote by $v: \overline {\varOmega }\times \overline {I}\to \mathbb {R}$ a sufficiently regular function that fulfills the Dirichlet boundary condition *v*=*g* on *Γ*_*D*_×*I* and the Signorini boundary condition *v*≥*g* on *Γ*_*S*_×*I*. Pointwise multiplication of the differential equation in (2.1) with *v*−*u*, integration over the spatial domain, and an application of Green’s identity yields

$$ \begin{aligned} &\int_{\varOmega } (\partial _{t} u - f) (v - u) + \int_{\varOmega } \bigl(\|\nabla u\|^{r-2} \nabla u \bigr) \cdot\nabla(v - u) \\ &\quad{}= \int_{\partial \varOmega } \bigl(\|\nabla u\|^{r-2} \partial _{ \mathfrak {n}} u (v - u) \bigr), \quad t \in I, \end{aligned} $$

where $\mathfrak {n}$ denotes the outer normal to the boundary and $\partial _{ \mathfrak {n}} u = \nabla u \cdot \mathfrak {n}$; to keep the formulae in a compact format, we occasionally do not indicate the dependence of the integrand on the spatial variable. Due to the fact that the contribution from the boundary term is non-negative, the evolutionary variational inequality

2.2 $$ \int_{\varOmega } (\partial _{t} u - f) (v - u) + \int_{\varOmega } \bigl(\|\nabla u\|^{r-2} \nabla u \bigr) \cdot\nabla(v - u) \geq0, \quad t \in I, $$

results.

### Underlying Function Spaces

The model problem suggests to consider the function spaces

$$ X = W^{1,r}(\varOmega ) \subset H = L^2(\varOmega ) \subset X^{*}= W^{-1,r^{*}}(\varOmega ) $$

as underlying Gelfand triple. The solution space is given by 
 and thus functions $f \in L^{r^{*}}(I,X^{*})$ defining the right-hand side of the differential equation are admitted. For a function *g*∈*X*=*W* ^1,*r*^(*Ω*) the imposed boundary conditions are incorporated in the (nonvoid) closed convex set

2.3 $$ K = \{w \in X: w = g \text{ a.e. on } \varGamma _{D}\text{ and } w \geq g \text{ a.e. on } \varGamma _{S}\}. $$

Due to the continuous embedding  the initial condition *u*(0)=*u*_0_ is well-defined. Furthermore, in order to ensure consistency with the boundary conditions, initial values *u*_0_∈*K* are considered.

### Governing Operators and Functionals

In regard to abstract evolutionary inequalities treated in Sect. 4, the first term in the variational inequality (2.2) corresponds to the duality pairing of *∂*_*t*_ *u*(⋅,*t*)−*f*(⋅,*t*)∈*X* ^∗^ and *v*(⋅,*t*)−*u*(⋅,*t*)∈*X*. The decisive second term is captured by the nonlinear functional $\varphi: X \times X \to \mathbb {R}$, for (fixed) *t*∈*I* defined through

2.4 $$ \varphi \bigl(u(\cdot,t),v(\cdot,t) \bigr) = \int _{\varOmega } \bigl(\bigl\Vert \nabla u(x,t)\bigr\Vert ^{r-2} \nabla u(x,t) \bigr) \cdot\nabla \bigl(v(x,t)-u(x,t) \bigr) \,\mathrm {d}x. $$

For the subsequent considerations it is essential that the functional *φ* is monotone-convex [32] and satisfies a growth condition, see Hypothesis 4.1 and [33, Sect. 10.3, Example 10.55] for detailed explanations. The monotonicity property *φ*(*u*,*v*)+*φ*(*v*,*u*)≤0 for all *u*,*v*∈*X* follows from elementary arguments for the associated real function $\widetilde {\varphi }(x,y) = \|x\|^{r-2} (x|y-x)$, where $x,y \in \mathbb {R}^{d}$. Besides, with the help of Young’s inequality the bound

$$ \bigl|\varphi(u,v) \bigr| \leq C \bigl(\|u\|_{X}^r + \|v-u\|_{X}^r \bigr), \quad u,v \in X, $$

follows.

### Connection to Nonlinear Evolution Equations

For the above model problem or, more generally, for problems governed by a monotone operator, the relation $\varphi(u,v) = \langle A u\vert v - u\rangle _{X^{*}\times X}$ establishes a connection between the nonlinear operator *A*:*X*→*X* ^∗^ corresponding to the weak formulation of the involved differential operator and the monotone-convex functional $\varphi: X \times X \to \mathbb {R}$, see also Sect. 4 for further details.

## Full Discretisation of the Signorini-Type Initial-Boundary Value Problem and Associated Discrete Variational Inequality

In the following, we introduce the considered full discretisations for nonlinear evolutionary inequalities. In Sect. 3.1 we specify the basic assumptions on the implicit Runge–Kutta time discretisations, and in Sect. 3.2 we treat the nonconforming *hp*-finite element space discretisations utilising the concept of set convergence of convex subsets in the sense of Glowinski–Mosco–Stummel. In order to deduce an appropriate fully discrete analogue to the variational inequality (2.2), we follow the arguments indicated in Sect. 2. In Sects. 4 and 5 the approach will be extended to more general nonlinear evolutionary inequalities.

### Time Discretisation by Stiffly Accurate Runge–Kutta Methods

Implicit Runge–Kutta methods such as Radau IIA methods are widely used in the context of stiff ordinary differential equations and parabolic evolution equations, primarily due to their favourable stability properties. However, to our knowledge, the present work is the first contribution where implicit Runge–Kutta time discretisations are studied for nonlinear evolutionary inequalities. In the following, we introduce the basic assumptions on the considered class of implicit Runge–Kutta methods and a fundamental auxiliary result. In order to state the defining relation for the time-discrete solution and its reformulation as time-discrete variational inequality, we find it useful to focus on the model problem and to utilise an approach via abstract evolutionary differential inclusions, as this resembles the approach for evolution equations exploited in [15].

In this section, for better readability and as the positive integer $M \in \mathbb {N}$ corresponding to the time discretisations are meanwhile fixed, we do not indicate the dependence of the quantities $\mathbb {I}$, *t*_*m*_, *τ* and *u*_*m*_ on *M*; the initial condition and its approximation are distinguished by the notations *u*(0) and *u*_0_, respectively.

#### Stiffly Accurate Runge–Kutta Methods

##### Stiffly Accurate Runge–Kutta Methods

We study implicit Runge–Kutta methods involving *s* stages, defined by a real matrix $\mathfrak {A}= (a_{ij})_{1 \leq i,j \leq s} \in \mathbb {R}^{s \times s}$, a vector of positive weights $\mathfrak {b}= (b_{i})_{1 \leq i \leq s} \in \mathbb {R}^{s}$ and a vector of associated nodes $\mathfrak {c}= (c_{i})_{1 \leq i \leq s} \in \mathbb {R}^{s}$ with 0<*c*_*i*_≤1 for 1≤*i*≤*s*. Throughout, we use the abbreviations $\alpha= \operatorname{diag} (a_{s1}, \dots, a_{ss}) \in \mathbb {R}^{s \times s}$ and $\gamma= \operatorname{diag} (c_{1}, \dots, c_{s}) \in \mathbb {R}^{s \times s}$; further, we set $\mathfrak {1}= (1,\dots,1)^{\mathrm{T}} \in \mathbb {R}^{s}$ and $\mathfrak {e}_{s} = (0,\dots,0,1)^{\mathrm{T}} \in \mathbb {R}^{s}$. We employ the following assumptions.

##### Hypothesis 3.1

(i) *The implicit Runge–Kutta method with coefficients given by* $( \mathfrak {A}, \mathfrak {c}, \mathfrak {b})$ *is stiffly accurate and consistent*, *that is*, *we have* *b*_*i*_=*a*_*si*_>0 *for* 1≤*i*≤*s* *and further* *a*_*i*1_+⋯+*a*_*is*_=*c*_*i*_ *for* 1≤*i*≤*s*, *in particular* *a*_*s*1_+⋯+*a*_*ss*_=*c*_*s*_=1.
(ii) *The coefficient matrix* $\mathfrak {A}$ *is invertible and the matrix*
  $$ \mathfrak {C}= \widetilde {\mathfrak {A}}+ \widetilde {\mathfrak {A}}^{\mathrm{T}} - \mathfrak {e}_s \mathfrak {e}_s^{\mathrm{T}} - ( \widetilde {\mathfrak {A}} \mathfrak {1}) (\widetilde {\mathfrak {A}} \mathfrak {1})^{\mathrm{T}} $$
  *is positive semi*-*definite*, *where* $\widetilde {\mathfrak {A}}= ( \widetilde {a}_{ij})_{1 \leq i,j \leq s} = \alpha \mathfrak {A}^{-1} \in \mathbb {R}^{s \times s}$.

##### Example Methods

The above hypotheses are in particular satisfied by the first-order implicit Euler method with *s*=*a*_11_=*c*_1_=1 and by the third-order two-stage Radau IIA method with coefficients

$$ a_{11} = \frac{5}{12}, \qquad a_{12} = - \frac{1}{12}, \qquad a_{21} = \frac{3}{4}, \qquad a_{22} = \frac{1}{4}, \qquad c_1 = \frac{1}{3}, \qquad c_2 = 1; $$

in both cases the matrix $\mathfrak {C}$ is equal to zero, and thus Hypothesis 3.1 implying algebraic stability [22] is obviously satisfied. More generally, as shown in [15], the *s*-stage Radau IIA method of order 2*s*−1 and the *s*-stage Lobatto IIIC method of order 2*s*−2 fulfill Hypothesis 3.1.

##### Basic Auxiliary Result

The following auxiliary result ensures that the first term in the employed discrete analogue of the variational inequality (2.2) defines a monotone-convex functional, see also [15, Lemma 3.4]. It generalises the relation

$$ x_1 (x_1 - x_0) = \frac{1}{2} \bigl(x_1^2 - x_0^2 \bigr) + \frac{1}{2} (x_1 - x_0)^2 \geq \frac{1}{2} \bigl(x_1^2 - x_0^2 \bigr), \quad x_0,x_1 \in \mathbb {R}, $$

known for the implicit Euler method. For the two- and three-stage Radau IIA methods analogous identities are established, whereas in the general case a lower bound is derived.

##### Lemma 3.1

*Hypothesis *3.1 *implies the following statements*.

(i) ([15, Lemma 3.4]) *For any* $\mathfrak {x}^{\mathrm{T}} = (x_{1}, \dots, x_{s}) \in \mathbb {R}^{s}$ *and* $x_{0} \in \mathbb {R}$ *we have*
  $$ \mathfrak {x}^{\mathrm{T}} \widetilde {\mathfrak {A}}( \mathfrak {x}- x_0 \mathfrak {1}) \geq \frac {1}{2} \bigl(x_s^2 - x_0^2 \bigr). $$
(ii)
  - *For the two*-*stage Radau IIA method the following identity is valid for arbitrary* $\mathfrak {x}^{\mathrm{T}} = (x_{1},x_{2}) \in \mathbb {R}^{2}$ *and* $x_{0} \in \mathbb {R}$:
    $$ \mathfrak {x}^{\mathrm{T}} \widetilde {\mathfrak {A}}( \mathfrak {x}- x_0 \mathfrak {1}) = \frac{1}{2} \bigl(x_2^2 - x_0^2 \bigr) + \frac{1}{2} \biggl(x_0 - x_1+ \frac {1}{2} (x_2 - x_1) \biggr)^2. $$
  - *For the three*-*stage Radau IIA method the following identity holds for any* $\mathfrak {x}^{\mathrm{T}} = (x_{1},x_{2},x_{3}) \in \mathbb {R}^{2}$ *and* $x_{0} \in \mathbb {R}$:
    $$ \begin{aligned}[c] \mathfrak {x}^{\mathrm{T}} \widetilde {\mathfrak {A}}( \mathfrak {x}- x_0 \mathfrak {1}) &= \frac{1}{2} \bigl(x_3^2 - x_0^2 \bigr) + \frac{1}{2} \biggl(x_0 - \biggl(\frac {1}{3} + \frac{1}{2} \sqrt{6} \biggr) x_1 \\ &\quad {}- \biggl(\frac{1}{3} - \frac {1}{2} \sqrt{6} \biggr) x_2 - \frac{1}{3} x_3 \biggr)^2. \end{aligned} $$

##### Proof

For the convenience of the reader, we indicate the proof of the first result found in [15].

(i) A straightforward calculation implies the following identity:

$$ \begin{gathered} \mathfrak {x}^{\mathrm{T}} \widetilde {\mathfrak {A}}( \mathfrak {x}- x_0 \mathfrak {1}) = \frac{1}{2} \bigl(x_s^2 - x_0^2 \bigr) + \frac{1}{2} \begin{pmatrix} x_0 & \mathfrak {x}^{\mathrm{T}} \end{pmatrix} \mathfrak {M}\begin{pmatrix} x_0 \\ \mathfrak {x}\end{pmatrix} , \\ \mathfrak {M}= \begin{pmatrix} 1 & - ( \widetilde {\mathfrak {A}} \mathfrak {1})^{\mathrm{T}} \\ - \widetilde {\mathfrak {A}}\mathfrak {1}& \widetilde {\mathfrak {A}}+ \widetilde {\mathfrak {A}}^{\mathrm{T}} - \mathfrak {e}_s \mathfrak {e}_s^{\mathrm{T}} \end{pmatrix} . \end{gathered} $$

A first Gauss elimination step applied to the symmetric matrix $\mathfrak {M}$ yields

$$ \mathfrak {M}= L_1 \begin{pmatrix} 1 & - ( \widetilde {\mathfrak {A}} \mathfrak {1})^{\mathrm{T}} \\ 0 & \mathfrak {C}\end{pmatrix} ; $$

applying the transposed transformation matrix from the right further implies

$$ \mathfrak {M}= L_1 \begin{pmatrix} 1 & 0 \\ 0 & \mathfrak {C}\end{pmatrix} L_1^{\mathrm{T}}. $$

By Hypothesis 3.1 the matrix $\mathfrak {C}$ is positive semi-definite, which implies positive semi-definiteness of $\mathfrak {M}$ and thus yields the statement.

(ii) (a) For the special case of the two-stage Radau IIA method Gauss elimination applied to $\mathfrak {M}$ leads to the matrix decomposition

$$ \begin{aligned} \mathfrak {M}= L D L^{\mathrm{T}}, \quad L = \begin{pmatrix} 1 & & \\ - \frac{3}{2} & 1 & \\ \frac {1}{2} & & 1 \end{pmatrix} ,\ D = \begin{pmatrix} 1 & & \\ & 0 & \\ & & 0 \end{pmatrix} . \end{aligned} $$

Employing the abbreviation $\mathfrak {y}^{\mathrm{T}} = (x_{0} \; \mathfrak {x}^{\mathrm{T}}) L = (x_{0} - x_{1}+ \frac{1}{2} (x_{2} - x_{1}), x_{1}, x_{2})$ the claimed result is obtained as follows:

$$ \begin{aligned} \mathfrak {x}^{\mathrm{T}} \widetilde {\mathfrak {A}}( \mathfrak {x}- x_0 \mathfrak {1}) &= \frac{1}{2} \bigl(x_2^2 - x_0^2 \bigr) + \frac{1}{2} \begin{pmatrix} x_0 & \mathfrak {x}^{\mathrm{T}} \end{pmatrix} L D L^{\mathrm{T}} \begin{pmatrix} x_0 \\ \mathfrak {x}\end{pmatrix} \\ &= \frac{1}{2} \bigl(x_2^2 - x_0^2 \bigr) + \frac{1}{2} \mathfrak {y}^{\mathrm{T}} D \mathfrak {y}\\ &= \frac{1}{2} \bigl(x_2^2 - x_0^2 \bigr) + \frac{1}{2} \biggl(x_0 - x_1+ \frac{1}{2} (x_2 - x_1) \biggr)^2. \end{aligned} $$

(b) Similar considerations yield the claimed relation for the three-stage Radau IIA method. □

##### Remark

We conjecture that analogous relations to Lemma 3.1(ii) are valid for Radau IIA methods of higher stage number. However, as in the present work our focus is on the derivation of a convergence result for stiffly implicit Runge–Kutta methods applied to evolutionary inequalities, but not on their rate of convergence, we do not further exploit this point. In view of Theorem 5.1 we note that under the above hypotheses the matrix $\widetilde {\mathfrak {A}}= \alpha \mathfrak {A}^{-1}$ is positive definite; in particular, for the two–stage Radau IIA method this follows from the stated relation (with *x*_0_=0) and elementary considerations.

#### Time Discretisation of Differential Inclusions

##### Differential Inclusions

In order to account for the introduction of stiffly accurate Runge–Kutta time discretisations for evolutionary inequalities (2.2), we employ an abstract formulation of the Signorini-type initial-boundary value problem (2.1) as differential inclusion

$$ u \in K, \quad\frac{\mathrm {d}}{\mathrm {d}t} u + A(u) + N_K(u) \ni f \quad \text{in } I, \quad u(0) \text{ given}. $$

Here, the nonlinear operator *A* is related to the *r*-Laplacian and the function *f* corresponds to the time-dependent right-hand side; furthermore, the set *K* as given in (2.3) and the normal cone map [4]

$$ u \in K \mapsto N_K(u), \quad N_K(u) = \bigl\{ w \in X^{*}: \langle w\vert v-u\rangle _{X^{*} \times X} \leq0 \text{ for all } v \in K \bigr\} , $$

capture the prescribed Dirichlet boundary condition and the unilateral constraint of the Signorini boundary condition.

##### Time-Discrete Solution

For an equidistant partition of the time interval

$$ \begin{gathered} \mathbb {I}: 0 = t_0 < \cdots< t_m < \cdots< t_{M} = T, \\ \tau = \frac{T}{M}, \qquad t_m = m \tau , \quad0 \leq m \leq M, \end{gathered} $$

and a given initial approximation *u*_0_≈*u*(0), the above differential inclusion motivates to determine approximation values *u*_*m*_≈*u*(*t*_*m*_) for 1≤*m*≤*M* through a recurrence relation of the form

$$ \left\{\begin{aligned} &\frac{1}{\tau } (U_{m-1,i} - u_{m-1} ) + \sum_{j=1}^{s} a_{ij} (A(U_{m-1,j}) + N_K(U_{m-1,j}) ) \\ &\quad{} \ni\sum_{j=1}^{s} a_{ij} F_{m-1,j},\quad1 \leq i \leq s , \\ &u_m = U_{m-1,s}, \end{aligned} \right. $$

where *F*_*m*−1,*i*_≈*f*(*t*_*m*−1_+*c*_*i*_ *τ*) denotes a given approximation of the right-hand side for 1≤*i*≤*s* and 1≤*m*≤*M*. Thus, at each time step, solving for the internal stages *U*_*m*−1_=(*U*_*m*−1,1_,…,*U*_*m*−1,*s*_)^T^ yields the new approximation value *u*_*m*_=*U*_*m*−1,*s*_. In compact matrix and vector notation, the relation for the stages can be rewritten as

3.1a $$ \frac{1}{\tau } (U_{m-1} - u_{m-1} \mathfrak {1}) + \mathfrak {A}A(U_{m-1}) + \mathfrak {A}N_K( U_{m-1}) \ni \mathfrak {A}F_{m-1}, $$

where *F*_*m*−1_=(*F*_*m*−1,1_,…,*F*_*m*−1,*s*_)^T^. Instead, we employ the formulation

3.1b $$ \frac{1}{\tau } \widetilde {\mathfrak {A}}(U_{m-1} - u_{m-1} \mathfrak {1}) + \alpha A(U_{m-1}) + N_K( U_{m-1}) \ni\alpha F_{m-1} $$

which will be justified in Sect. 3.1.3; note that *αN*_*K*_(*U*_*m*−1_)=*N*_*K*_(*U*_*m*−1_), due to the positivity of the weights.

#### Time-Discrete Variational Inequality

##### Time-Discrete Solution to Model Problem

The application of a stiffly accurate Runge–Kutta method to the Signorini-type initial-boundary value problem (2.1) yields

$$ \frac{1}{\tau } \sum_{j=1}^{s} \widetilde {a}_{ij} (U_{m-1,j} - u_{m-1}) - \alpha_i \operatorname{div} \bigl(\|\nabla U_{m-1,i} \|^{r-2} \nabla U_{m-1,i} \bigr) = \alpha_i F_{m-1,i}\quad \text{in } \varOmega , $$

where the stages are subject to the Dirichlet boundary conditions *U*_*m*−1,*i*_=*g* on *Γ*_*D*_ and the unilateral constraints *U*_*m*−1,*i*_≥*g*, $\|\nabla U_{m-1,i}\|^{r-2} \partial _{ \mathfrak {n}} U_{m-1,i} \geq0$, $(U_{m-1,i} - g) \|\nabla U_{m-1,i}\|^{r-2} \partial _{ \mathfrak {n}} U_{m-1,i} = 0$ on *Γ*_*S*_, for all 1≤*i*≤*s* and 1≤*m*≤*M*, see (3.1b). Under these constraints on *U*_*m*−1,*i*_ for 1≤*i*≤*s* and 1≤*m*≤*M*, the above formulation is equivalent to

$$ \frac{1}{\tau } (U_{m-1,i} - u_{m-1}) - \sum _{j=1}^{s} a_{ij} \operatorname{div} \bigl( \|\nabla U_{m-1,j}\|^{r-2} \nabla U_{m-1,j} \bigr) = \sum_{j=1}^{s} a_{ij} F_{m-1,j} \quad \text{in } \varOmega $$

which corresponds to (3.1a).

##### Time-Discrete Variational Inequality

In order to deduce an appropriate time-discrete variational inequality, we imitate the procedure for the time-continuous problem. Suppose *V*_*m*−1,*i*_∈*K* and set *v*_*m*_=*V*_*m*−1,*s*_. Testing the differential equation with *V*_*m*−1,*i*_−*U*_*m*−1,*i*_ and applying integration by parts implies

$$ \begin{aligned} &\int_{\varOmega } \Biggl( \frac{1}{\tau } \sum_{j=1}^{s} \widetilde {a}_{ij} (U_{m-1,j} - u_{m-1}) - \alpha_i F_{m-1,i} \Biggr) (V_{m-1,i} - U_{m-1,i}) \\ &\quad{}+ \alpha_i \int_{\varOmega } \bigl(\|\nabla U_{m-1,i}\|^{r-2} \nabla U_{m-1,i} \bigr) \cdot \nabla(V_{m-1,i} - U_{m-1,i}) \geq0 ; \end{aligned} $$

we point out that it is essential to employ the reformulation (3.1b) instead of (3.1a), since it is then evident that the boundary term is non-negative, due to boundary conditions and the positivity of the weights *α*_*i*_>0 for 1≤*i*≤*s*. Finally, summation of the relations for 1≤*i*≤*s* yields an appropriate time-discrete analogue of (2.2) for any 1≤*m*≤*M*

3.2a $$ \begin{aligned} &\sum_{i=1}^{s} \int_{\varOmega } \Biggl(\frac{1}{\tau } \sum _{j=1}^{s} \widetilde {a}_{ij} ( U_{m-1,j} - u_{m-1}) - \alpha_i F_{m-1,i} \Biggr) (V_{m-1,i} - U_{m-1,i}) \\ &\quad{}+ \sum_{i=1}^{s} \alpha_i \int_{\varOmega } \bigl(\|\nabla U_{m-1,i}\|^{r-2} \nabla U_{m-1,i} \bigr) \cdot\nabla(V_{m-1,i} - U_{m-1,i}) \geq 0 ; \end{aligned} $$

in compact notation, we thus obtain

3.2b $$ \begin{aligned} &\int_{\varOmega } (V_{m-1} - U_{m-1})^{\mathrm{T}} \biggl(\frac {1}{\tau } \widetilde {\mathfrak {A}}( U_{m-1} - u_{m-1} \mathfrak {1}) - \alpha F_{m-1} \biggr) \\ &\quad{}+ \sum_{i=1}^{s} \alpha_i \varphi (U_{m-1,i},V_{m-1,i} ) \geq0 \end{aligned} $$

with the functional *φ* defined in (2.4).

##### Governing Functionals

It is essential that the term

$$ \frac{1}{\tau } \int_{\varOmega } (V_{m-1} - U_{m-1})^{\mathrm{T}} \widetilde {\mathfrak {A}}(U_{m-1} - u_{m-1} \mathfrak {1}) $$

which results from the employed reformulation (3.1b) and summation, defines a monotone-convex functional; this follows from the fact that the associated real function defined by $\widetilde {\psi }( \mathfrak {x}, \mathfrak {y}) = ( \mathfrak {y}- \mathfrak {x})^{\mathrm{T}} \widetilde {\mathfrak {A}}( \mathfrak {x}- x_{0} \mathfrak {1})$ for $\mathfrak {x}, \mathfrak {y}\in \mathbb {R}^{s}$ and $x_{0} \in \mathbb {R}$ is monotone-convex, since the relation $\widetilde {\psi }( \mathfrak {x}, \mathfrak {y}) + \widetilde {\psi }( \mathfrak {y}, \mathfrak {x}) = - ( \mathfrak {x}- \mathfrak {y})^{\mathrm{T}} \widetilde {\mathfrak {A}}( \mathfrak {x}- \mathfrak {y}) \leq0$ is ensured by Lemma 3.1. Provided that the functional *φ* is monotone-convex, it is evident that the second integral involving the positive weights *α*_*i*_ for 1≤*i*≤*s* defines a monotone-convex functional.

### Space Discretisation by the *hp*-Finite Element Method

In the following, we introduce approximations based on the *hp*-finite element method and the concept of Glowinski–Mosco–Stummel set convergence of convex subsets. For our considerations in the subsequent sections, it is convenient to employ a general setting of nonconforming Galerkin methods. Finally, we state the fully discrete analogue of the evolutionary variational inequality, which gives rise to a finite-dimensional and hence computable problem.

#### Approximations Based on the *hp*-Finite Element Method

##### Basic Assumptions

For simplicity and as this is no restriction of generality, we consider a polygonal planar domain $\varOmega \subset \mathbb {R}^{2}$; in fact, the *p*- and *hp*-finite element approximation on curvilinear domains is well-understood, see [5], and the analysis to follow can be extended to higher dimensional domains by tensor product approximation. Moreover, we assume that there is only a finite number of *end points* $\overline {\varGamma }_{D}\cap \overline {\varGamma }_{S}$, which guarantees the density relation , see [25]. For ease of notation we do not indicate the dependence of the quantities *h*,*i*,*p*,*q*,*G*,ℰ on the positive integer $N \in \mathbb {N}$.

##### Finite Element Subspaces

We let  be a sequence of shape regular meshes, see [34] that covers the bounded polygonal domain $\varOmega \subset \mathbb {R}^{2}$ and consists of affine quadrilaterals  with diameter *h*_*Q*_ such that all corners of the boundary *Γ* and all end points $\overline {\varGamma }_{D}\cap \overline {\varGamma }_{S}$ are nodes of the mesh . Obviously, for every edge *E* of  there exists a unique element  such that *E* is an edge of *Q*_*E*_. For each affine quadrilateral  we denote by $p_{Q} \in \mathbb {N}$ the associated polynomial degree, assuming that neighbouring elements have comparable polynomial degrees, that is, there exists a constant *c*>0 such that the relation *c* ^−1^ *p*_*Q*_≤*p*_*Q*′_≤*cp*_*Q*_ holds for elements  with $\overline {Q} \cap \overline {Q'} \neq\emptyset$. Moreover, we denote by *Π*_*p*_(*Q*) the tensor product space of polynomials of degree *p* on *Q* and define the finite element subspaces $(X^{(N)})_{N \in \mathbb {N}}$ through 
3.3a


##### Gauss–Lobatto Quadrature

Similar to [11, 12, 28] we employ Gauss–Lobatto quadrature in the discretisation procedure. To this end, we introduce the Gauss–Lobatto quadrature nodes (*ξ*_*j*_)_0≤*j*≤*q*_, given as zeros of the function $(1- \xi^{2}) L_{q}' ( \xi)$, where *L*_*q*_ denotes the Legendre polynomial of degree *q*≥1. Obviously, the nodes *ξ*_0_=−1 and *ξ*_*q*_=1 coincide with the end points of the reference interval [−1,1]. As well-known, there exist positive weights such that the associated quadrature formula is exact for all polynomials *ϕ* up to degree 2*q*−1,

$$ \int_{-1}^1 \phi(\xi) \, \mathrm {d}\xi= \sum _{j=0}^{q} \omega_j \phi (\xi _j), \quad\omega_j = \frac{1}{q (q+1) (L_q(\xi_j) )^2}, $$

see for instance [6, Chap. I, Sect. 4].

##### Interpolation Operators

For any edge *E* of a mesh  we introduce the quadrature order $q_{E} = p_{Q_{E}}$, and by affine transformation $F_{E}: [-1,1] \rightarrow\overline{E}$ we define the set *G*_*E*_ of *q*_*E*_+1 associated Gauss–Lobatto nodes. Local and global interpolation operators associated with the Gauss–Lobatto nodes are thus given by 



##### Convex Subsets

In addition, we introduce the sets of edges on the Dirichlet and Signorini boundary, respectively, 
 which provides the associated sets of Gauss–Lobatto nodes 
 Choosing the Gauss–Lobatto nodes as control points of the boundary conditions, we define a family of closed convex subsets $(K^{(N)})_{N \in \mathbb {N}}$ by

3.3b $$ K^{(N)}= \bigl\{ w^{(N)}\in X^{(N)}: w^{(N)}= g \text{ on } G_{D} \text{ and } w^{(N)} \geq g \text{ on } G_{S} \bigr\} . $$

We note that the approximation is nonconforming, since the subset *K* ^(*N*)^ is generally not contained in the set *K*, in particular, for polynomial degree ≥2 or a non-convex obstacle *g*. Instead we have

$$ \bigcap_{N \in \mathbb {N}} K^{(N)}\cap K \neq\emptyset, $$

and we are able to prove set convergence, see Lemma 3.3.

##### Interpolation Operators

Likewise, local and global interpolation operators associated with pairs of Gauss–Lobatto nodes *G*_*Q*_={(*ξ*_*i*_,*ξ*_*j*_)|0≤*i*,*j*≤*p*_*Q*_} and the affine transformation $F_{Q}: [-1,1]^{2} \rightarrow \overline {Q}$, are given by 



##### Polynomial Interpolation Error

For later use we recall the following result on the polynomial interpolation error in the reference interval $\widehat {E} = (-1, 1)$ and the reference square $\widehat {Q} = (-1,1)^{2}$, respectively.

##### Theorem 3.2

([6, Theorems 13.4, 14.2])

(i) *For any pair of real numbers* $(r,s) \in \mathbb {R}^{2}$ *satisfying* 0≤*r*≤1 *and* $s > \frac{1+r}{2}$, *there exists a positive constant* *c*=*c*(*s*)>0 *depending only on s* *such that for any function* $\eta\in H^{s}(\widehat {E})$ *the following estimate holds*
  $$ \|\eta- i_{\widehat {E},q} \eta\|_{H^r(\widehat {E})} \leq c q^{r-s} \|\eta\|_{H^s(\widehat {E})}. $$
(ii) *For any pair of real numbers* $(r,s) \in \mathbb {R}^{2}$ *satisfying* 0≤*r*≤1 *and* $s > \frac{1+r}{2}$, *there exists a positive constant* *c*=*c*(*s*)>0 *depending only on s* *such that for any function* $\psi\in H^{s}(\widehat {Q})$ *the following relation is valid*
  $$ \|\eta- i_{\widehat {Q},p} \psi\|_{H^r(\widehat {Q})} \leq c p^{r-s} \|\psi\|_{H^s(\widehat {Q})}. $$

#### Set Convergence

##### Set Convergence

We employ the notion of Glowinski–Mosco–Stummel set convergence of convex subsets, introduced in [29, 38], further analysed in [3] and refined in [17, 25]. Provided that the following conditions are satisfied, the sequence $(K^{(N)} )_{N \in \mathbb {N}}$ is said to *G*-converge to the set *K* for *N*→∞, that is,

$$ K^{(N)}\overset{N \to\infty}{\underset{G}{\longrightarrow}} K. $$

(i) For any subsequence $(N_{\ell})_{\ell\in \mathbb {N}}$ such that *N*_*ℓ*_→∞ for *ℓ*→∞ and for any sequence $(x_{N_{\ell }})_{\ell\in \mathbb {N}}$ such that $x_{N_{\ell}} \in K^{(N_{\ell})}$ and $(x_{N_{\ell}})_{\ell\in \mathbb {N}}$ converges to *x* for *ℓ*→∞, weakly in *X*, it follows *x*∈*K*.
(ii) There exist a dense subset $\widetilde {K} \subset K$ and mappings $\varrho ^{(N)}: \widetilde {K} \to K^{(N)}$ for $N \in \mathbb {N}$ such that for any element $x \in \widetilde {K}$ the sequence $(\varrho^{(N)}(x))_{N \in \mathbb {N}}$ converges to *x* for *N*→∞, strongly in *X*, and further we have *ϱ* ^(*N*)^(*x*)∈*K* ^(*N*)^ for all *N*≥*N*_0_(*x*) with $N_{0}(x) \in \mathbb {N}$.

As shown in [3, 20], e.g., *G*-convergence of sets gives rise to convergence (sometimes called epiconvergence) of convex lower semi-continuous functionals, when considering their epigraphs, which are convex closed sets. The following result ensures set convergence of the sequence $(K^{(N)})_{N \in \mathbb {N}}$ defined in (3.3a), (3.3b) towards *K*, see also (2.3) for the definition of *K*.

##### Lemma 3.3

*Assume that for the polygonal domain* $\varOmega \subset \mathbb {R}^{2}$ *there exist only a finite number of end points* $\overline {\varGamma }_{D}\cap \overline {\varGamma }_{S}$ *and that the gap function*  *belongs to* $H^{\frac{1}{2} + \varepsilon}( \varGamma _{S})$ *for some* *ε*>0. *Then*, *provided that*

*the sequence* $(K^{(N)})_{N \in \mathbb {N}}$ *G*-*converges to K* *for* *N*→∞.

##### Proof

Classical *h*-FEM convergence for a similar variational problem is already treated in [19], where Newton–Cotes formulae are used instead of Gauss–Lobatto quadrature. Inspecting the proof of [19, Theorem 4.1] shows that the norm convergence for a fixed quadrature order hinges on the positiveness of the quadrature weights, what is satisfied for all quadrature orders with Gauss–Lobatto quadrature. Therefore in the following we may focus on the case where *h*_*Q*_ is fixed for all  and . Moreover, as every equality constraint *w*=*g* can be expressed through the two inequality constraints *w*≤*g* and *w*≥*g*, it suffices to treat the Signorini boundary condition.

In order to verify the first requirement of *G*-convergence we have to show that for any  with $\lambda\vert _{ \varGamma _{S}} \geq0$ it follows that

3.4 $$ \int_{ \varGamma _{S}} (w - g) \lambda\, \mathrm {d}x\leq0, $$

using duality with respect to (*L* ^1^,*L* ^∞^). Moreover, since the mesh  is supposed to be independent of *N*, we can simply consider the above integral on any fixed edge *E*. Thus, we fix  with *λ*≥0 and also the polynomial degree *q*=*q*_*E*_. Similarly as [28] we approximate the function *λ* by a combination of Bernstein polynomials *B*_*q*_, and with the local mapping $F_{E}: [-1,1] \rightarrow\overline{E}$ we define first on [−1,1],

$$ \tilde{\lambda}_q ( t) = (B_q \lambda\circ F_E) (t) = \sum_{k=0}^q \tbinom{q}{k} \biggl(\frac{1+t}{2}\biggr)^k \biggl( \frac {1-t}{2}\biggr)^{q-k} (\lambda\circ F_E ) \biggl( \frac{2k}{q} - 1\biggr) $$

and then on *E*,

$$\lambda_q (x) = \tilde{\lambda}_q \bigl( F_E^{-1}x \bigr) . $$

As the Bernstein operators are monotone, it follows *λ*_*q*_≥0. By [10, Chap. 1, Theorem 2.3] we further have

3.5 $$ \lim_{q \rightarrow\infty} \| \lambda_q - \lambda \|_{L^\infty(E)} = 0. $$

Next, we introduce the interpolant *g* ^(*N*)^=*i*_*E*,*q*_ *g* of the gap function , and we obtain from Theorem 3.2(i) with *r*=0 and $s = \frac{1}{2} + \varepsilon$ the relation

$$ \lim_{N \rightarrow\infty} \bigl\| g^{(N)}- g\bigr\| _{L^2(E)} = 0. $$

Since the trace map *W* ^1,*r*^(*Ω*)→*L* ^1^(*E*) is weakly continuous, *w* ^(*N*)^⇀*w* in *L* ^1^(*E*) and $\|w^{(N)} \|_{L^{1}(E)}$ is bounded. Therefore setting *q*=*q*_*E*_, by means of the estimate

$$ \begin{aligned} &\biggl|\int_E \bigl( \bigl(w^{(N)}- g^{(N)} \bigr) \lambda_{q - 1} - (w - g) \lambda \bigr) \, \mathrm {d}x\biggr| \\ &\quad{}\leq \bigl\| w^{(N)}- g^{(N)}\bigr\| _{L^1(E)} \|\lambda_{q - 1} - \lambda\|_{L^\infty (E)} \\ &\qquad {}+ \biggl|\int_E \bigl( \bigl(w^{(N)}- g^{(N)} \bigr) - (w - g) \bigr) \lambda\, \mathrm {d}x\biggr|, \end{aligned} $$

relation (3.5) and *λ*∈*L* ^∞^(*E*)=(*L* ^1^(*E*))^∗^, we conclude

3.6 $$ \lim_{N \rightarrow\infty} \int_E \bigl(w^{(N)}- g^{(N)}\bigr) \lambda_{q- 1} \, \mathrm {d}x= \int_E (w - g) \lambda\, \mathrm {d}x. $$

Using on the other hand that *λ*_*q*−1_ (*w* ^(*N*)^−*g* ^(*N*)^)|_*E*_ is a polynomial of degree 2*q*−1 and that hence the above integral can be evaluated exactly by the Gauss–Lobatto quadrature we obtain for

$$f = \bigl( \bigl(w^{(N)}- g^{(N)} \bigr) \lambda_{q -1} \bigr) \circ F_E $$

that

$$ \int_E \bigl(w^{(N)}- g^{(N)} \bigr) \lambda_{q_{E} - 1} \, \mathrm {d}x= \sum_{j=0}^q \omega_j f(\xi_j) . $$

Due to the fact that the quadrature weights *ω*_*j*_>0 are positive and further *λ*_*q*−1_≥0 as well as ((*w* ^(*N*)^−*g* ^(*N*)^)∘*F*_*E*_)(*ξ*_*j*_)≤0 as *w* ^(*N*)^∈*K* ^(*N*)^, we arrive at

$$ \int_E \bigl(w^{(N)}- g^{(N)} \bigr) \lambda_{q_{E} - 1} \, \mathrm {d}x\leq0. $$

In view of (3.6) this proves our claim (3.4).

It remains to prove the second requirement. By the finiteness assumption, due to [25] it follows that  is dense in *K*. Consequently, we may consider the dense subset  and may define *ϱ* ^(*N*)^ as the Lagrange interpolation operator on $\widetilde {K}$ in *X* ^(*N*)^. Moreover, as $w \in \widetilde {K}$ satisfies the constraints in *K* pointwise, we have *ϱ* ^(*N*)^ *w*∈*K* ^(*N*)^ for all $w \in \widetilde {K}$. By Theorem 3.2(ii) it follows *ϱ* ^(*N*)^ *w*→*w* as *N*→∞ in *W* ^1,*r*^(*Ω*). Altogether, this yields the stated result. □

#### Fully Discrete Variational Inequality

##### Assumptions on Approximating Spaces

In accordance with Sects. 3.2.1 and 3.2.2, we henceforth employ the following assumptions on the general Galerkin method that can be realized by the *hp*-finite element method.

##### Hypothesis 3.2

(i) *For the Banach space X* *there exists a sequence* $(X^{(N)})_{N \in \mathbb {N}}$ *of finite*-*dimensional subspaces of X* *such that*
  $$ X = \overline{\bigcup_{N \in \mathbb {N}} X^{(N)}} $$
  *with respect to *∥⋅∥_*X*_.
(ii) *For the Hilbert space H* *there exists a sequence* $(H^{(N)})_{N \in \mathbb {N}}$ *of finite*-*dimensional subspaces of H* *with* *X* ^(*N*)^⊂*H* ^(*N*)^ *such that*
  $$ H = \overline{\bigcup_{N \in \mathbb {N}} H^{(N)}} $$
  *with respect to *∥⋅∥_*H*_.
(iii) *For the closed convex set K* *there exists a sequence* $(K^{(N)})_{N \in \mathbb {N}}$ *of closed convex nonvoid subsets* *K* ^(*N*)^⊂*X* ^(*N*)^ *such that*
  $$ \bigcap_{N \in \mathbb {N}} K^{(N)}\cap K \neq\emptyset $$
  *with respect to *∥⋅∥_*H*_.

##### Fully Discrete Variational Inequality

The spatial discretisation of the time-discrete variational inequality (3.2a), (3.2b) by the Galerkin approach yields the following fully discrete variational inequality: Find $(U^{(M,N)}_{m-1})_{1 \leq m \leq M}$ with $U^{(M,N)}_{m-1,i} \in K^{(N)}$ for 1≤*i*≤*s* and 1≤*m*≤*M* such that

3.7 $$\begin{aligned} &\int_{\varOmega } \bigl(V^{(M,N)}_{m-1} - U^{(M,N)}_{m-1} \bigr)^{\mathrm{T}} \biggl(\frac {1}{\tau^{(M)} } \widetilde {\mathfrak {A}}\bigl(U^{(M,N)}_{m-1} - u^{(M,N)}_{m-1} \mathfrak {1}\bigr) - \alpha F^{(M,N)} _{m-1} \biggr) \\ &\quad{}+ \sum_{i=1}^{s} \alpha_i \varphi \bigl(U^{(M,N)}_{m-1,i},V^{(M,N)} _{m-1,i} \bigr) \geq0 \end{aligned}$$

for all $V^{(M,N)}_{m-1,i} \in K^{(N)}$ with 1≤*i*≤*s* and 1≤*m*≤*M*, where $u^{(M,N)}_{0} = u^{(N)}_{0} \in K^{(N)}$ as well as $F^{(M,N)} _{m-1,i}$ are given approximations and $u^{(M,N)}_{m} = U^{(M,N)}_{m-1,s}$ for 1≤*i*≤*s* and 1≤*m*≤*M*, see also (2.2).

## Nonlinear Evolutionary Variational Inequalities

In Sects. 2 and 3 the focus is on a specific application, the Signorini-type initial-boundary value problem (2.1), its formulation as evolutionary variational inequality (2.2) and the derivation of the fully discrete analogue (3.7). In this section, we introduce a general framework of nonlinear evolutionary variational inequalities involving monotone-convex functionals. Furthermore, following [8] we introduce an integrated and relaxed reformulation of the problem and state a uniqueness result.

### Analytical Framework

#### Underlying Function Spaces and Governing Functional

The underlying Gelfand triple *X*⊂*H*⊂*X* ^∗^, with continuous and dense embeddings, is formed by the Banach space *X*, its dual space *X* ^∗^ and the Hilbert space *H*, as usual identified with its dual space. Basic assumptions on the governing nonlinear functional are collected in the following hypotheses.

#### Hypothesis 4.1

*Let* *r*∈[2,∞) *and* $\varphi: X \times X \to \mathbb {R}$.

(i) *For any* *x*∈*X* *the relation* *φ*(*x*,*x*)=0 *holds*.
(ii) Convexity. *For any* *z*∈*X* *the function* $\psi= \varphi(z,\cdot): X \to \mathbb {R}$ *is convex*, *that is*, *for all* *x*,*y*∈*X* *and* *σ*∈[0,1] *we have*
  $$ \psi \bigl((1-\sigma) x + \sigma y \bigr) \leq(1-\sigma) \psi(x) + \sigma \psi (y). $$
(iii) Sequential lower semi-continuity. *For any* *z*∈*X* *the function* $\psi= \varphi(z,\cdot): X \to \mathbb {R}$ *is sequentially lower semi*-*continuous*, *that is*, *for every* *x*∈*X* *and for any sequence* $(x_{k})_{k \in \mathbb {N}}$ *in X* *with* *x*_*k*_→*x* *as* *k*→∞ *we have*
  $$ \liminf_{k \in \mathbb {N}} \psi(x_k) \geq\psi(x). $$
(iv) Hemi-continuity. *The functional* $\varphi: X \times X \to \mathbb {R}$ *is hemi*-*continuous*, *that is*, *for all* $x,\widetilde {x},y \in X$ *the mapping*
  $$ [0,1] \longrightarrow \mathbb {R}: \sigma\longmapsto\varphi(x + \sigma \widetilde{x},y) $$
  *is continuous*.
(v) Monotonicity. *The functional* $\varphi: X \times X \to \mathbb {R}$ *is monotone*, *that is*, *for all* *x*,*y*∈*X* *we have*
  $$ \varphi(x,y) + \varphi(y,x) \leq0. $$
(vi) Coercivity. *The functional* $\varphi: X \times X \to \mathbb {R}$ *is coercive*, *that is*, *there exist constants* *C*>0 *and* *C*_0_≥0 *such that for all* *x*∈*X*
  $$ \varphi(x,0) \leq C_0 - C \|x\|_{X}^r. $$
(vii) Growth condition. *There exists a constant* *C*>0 *such that the operator* $\varphi: X \times X \to \mathbb {R}$ *satisfies the following growth condition for any* *x*,*y*∈*X*:
  $$ \bigl|\varphi(x,y) \bigr| \leq C \bigl(1 + \|x\|_{X}^{r-1} \bigr) \|y-x\|_{X}. $$

#### Connection to Nonlinear Evolution Equations

The above hypotheses on the functional *φ* are in accordance with the model problem (2.1) governed by the *r*-Laplacian or, more generally, evolutionary problems governed by a nonlinear operator *A*:*X*→*X* ^∗^ that is hemi-continuous, monotone, coercive and satisfies a certain growth condition, see for instance [15] and references given therein. In this case, the functional

$$ \varphi: X \times X \longrightarrow \mathbb {R}: (x,y) \longmapsto\varphi (x,y) = \langle A x\vert y - x\rangle _{X^{*}\times X} $$

inherits the properties of *A*. Sequential lower semi-continuity and hemi-continuity are evident; moreover, monotonicity, coercitivity and a certain growth condition hold.

- Provided that the operator *A* is monotone, that is, for all *x*,*y*∈*X* the relation $\langle A y - A x\vert y - x\rangle _{X^{*}\times X} \geq0$ holds, the associated functional *φ* is monotone, since $\varphi(x,y) + \varphi(y,x) = - \langle A y - A x\vert y - x\rangle _{X^{*}\times X} \leq0$ for all *x*,*y*∈*X*.
- If *A* is coercive with exponent *r*∈[2,∞), that is, for any *x*∈*X* a bound of the form $\langle A x\vert x\rangle _{X^{*}\times X} \geq C \|x\|_{X}^{r} - C_{0}$ holds with constants *C*>0 and *C*_0_≥0, the associated functional *φ* is coercive, since $- \varphi(x,0) = \langle A x\vert x\rangle _{X^{*}\times X} \geq C \|x\|_{X}^{r} - C_{0}$ and thus $\varphi (x,0) \leq C_{0} - C \|x\|_{X}^{r}$ for all *x*∈*X*.
- Finally, provided that the operator *A* satisfies the growth condition $\|A x\|_{X^{*}} \leq C (1 + \|x\|_{X}^{r-1})$ for all *x*∈*X*, the associated functional *φ* fulfills the bound $|\varphi (x,y)| \leq \|A x\|_{X^{*}} \|y - x\|_{X} \leq C (1 + \|x\|_{X}^{r-1} ) \|y - x\|_{X}$ for any *x*,*y*∈*X*.

These conditions are also satisfied for functionals given by $\varphi(x,y) = \langle A x\vert y - x\rangle _{X^{*}\times X} + f(y) - f(x)$, provided that additionally $f: X \to \mathbb {R}$ is convex and lower semi-continuous.

#### Related Functionals and Operators

Under the assumptions of Hypothesis 4.1, the growth condition on *φ* and an application of Young’s inequality yield the estimate

$$ \bigl|\varphi(x,y) \bigr| \leq C \bigl(1 + \|x\|_{X}^{r} + \|y-x\|_{X}^{r} \bigr), \quad x,y \in X. $$

A sufficient condition for measurability of the function $I \to \mathbb {R}: t \mapsto\varphi(x(t),y(t))$, where *x*,*y*:*I*→*X* are assumed to be Bochner integrable, is that *φ* is a Caratheodory function, which here amounts to continuity of (*x*,*y*)↦*φ*(*x*,*y*) with respect to the norm topology, see also [2]. As a consequence, the Nemytskii operator associated with the nonlinear functional *φ* and a related functional,

4.1 $$ \begin{gathered} L^r(I,X) \times L^r(I,X) \rightarrow L^1(I, \mathbb {R}): (x,y) \mapsto\varphi (x,y), \\ \varPhi : L^r(I,X) \times L^r(I,X) \to \mathbb {R}: (x,y) \mapsto\int _{0}^{\mathrm{T}} \varphi \bigl(x(t),y(t) \bigr) \, \mathrm {d}t, \end{gathered} $$

are well-defined, since the bound

$$ \begin{aligned} \bigl|\varPhi (x,y) \bigr| &\leq \bigl\| \varphi(x,y)\bigr\| _{L^1(I, \mathbb {R})} = \int_{0}^{\mathrm{T}} \bigl|\varphi \bigl(x(t),y(t) \bigr) \bigr| \, \mathrm {d}t \\ &\leq C \biggl(1 + \int_{0}^{\mathrm{T}} \bigl\| x(t)\bigr\| _{X}^r \, \mathrm {d}t + \int_{0}^{\mathrm{T}} \bigl\| y(t)-x(t)\bigr\| _{X}^r \, \mathrm {d}t \biggr) \\ &\leq C \bigl(1 + \|x\|_{L^r(I,X)}^r + \|y-x\|_{L^r(I,X)}^r \bigr) < \infty \end{aligned} $$

is ensured for all *x*,*y*∈*L* ^*r*^(*I*,*X*). Moreover, the functional *Φ* inherits the property of hemi-continuity, see [8] for further details. For the derivation of the main result, an enhancement of sequential lower semi-continuity is needed.

#### Hypothesis 4.2

*The functional Φ* *defined in *(4.1) *is continuous with respect to the second argument and further satisfies the LSC condition*, *that is*, *for all sequences* $(x_{j})_{j \in \mathbb {N}}$ *and* $(y_{j})_{j \in \mathbb {N}}$ *such that x*_*j*_ *converges to x* *for* *j*→∞, *strongly in L* ^*r*^(*I*,*X*) *and y*_*j*_ *converges to y* *for* *j*→∞, *weakly in L* ^*r*^(*I*,*X*), *it follows that*

$$ \liminf_{j \to\infty} \varPhi (x_j,y_j) \geq \varPhi (x,y). $$

#### Solution Space

In the following, the Banach space 
 which is continuously embedded in the space of continuous functions , will be the solution space, and a nonvoid closed convex set *K*⊂*X* shall capture the imposed boundary conditions. Throughout, we employ the abbreviations 
 and utilise the continuous embedding . For the problem data, the function defining the right-hand side and the initial value, we suppose $f \in L^{r^{*}}(I,X^{*})$ and *u*_0_∈*K*. For the sake of brevity, we do not indicate the dependence of  and  on the considered time interval *I*, the exponent *r*∈[2,∞) given by Hypothesis 4.1, the associated exponent $r^{*}= \frac{r}{r-1} \in(1,2]$ and the underlying Gelfand triple.

### Evolutionary Inequality and Reformulations

#### Nonlinear Evolutionary Variational Inequality

The formulation of the model problem (2.1) as evolutionary variational inequality (2.2) motivates the following general form of a nonlinear evolutionary variational inequality involving a nonlinear convex-monotone functional, see also Sect. 4.1 for the hypotheses on *φ* and the definition of the solution space.

#### Problem 4.1

*For a given initial value* *u*_0_∈*K* *and a given function* $f \in L^{r^{*} }(I,X^{*})$ *find*  *such that* *u*(0)=*u*_0_ *and*

4.2 $$ \biggl\langle \frac{\mathrm {d}}{\mathrm {d}t} u(t) - f(t) \bigg\vert v(t) - u(t) \biggr\rangle _{X^{*}\times X} + \varphi \bigl(u(t), v(t) \bigr) \geq0 $$

*for* *v*(*t*)∈*K* *and almost all* *t*∈*I*.

#### Integrated Formulation of the Evolutionary Inequality

Integration over the time domain yields the following formulation, which is equivalent to (4.2) under Hypothesis 4.1, see [8, Lemma 2.3].

#### Problem 4.2

*For given* *u*_0_∈*K* *and* $f \in L^{r^{*}}(I,X^{*})$ *find*  *such that* *u*(0)=*u*_0_ *and*

4.3 $$ \int_{0}^{\mathrm{T}} \biggl\langle \frac{\mathrm {d}}{\mathrm {d}t} u(t) - f(t) \bigg\vert v(t) - u(t) \biggr\rangle _{X^{*}\times X} \, \mathrm {d}t + \varPhi (u, v) \geq0 $$

*for all* *v*∈*L* ^*r*^(*I*,*K*), *where the functional Φ* *is defined by *(4.1).

#### Relaxed Formulation of the Evolutionary Inequality

A reformulation of (4.2) which presupposes reduced regularity requirements on the solution is as follows.

#### Problem 4.3

*For given* *u*_0_∈*K* *and* $f \in L^{r^{*}}(I,X^{*})$ *find*  *such that* *u*(0)=*u*_0_ *and*

4.4 $$ \int_{0}^{\mathrm{T}} \biggl\langle \frac{\mathrm {d}}{\mathrm {d}t} v(t) - f(t) \bigg\vert u(t) - v(t) \biggr\rangle _{X^{*}\times X} \, \mathrm {d}t + \varPhi (v, u) \leq\frac{1}{2} \bigl\| v(0) - u_0\bigr\| _{H}^2 $$

*for all* .

Any solution to the integrated variational inequality is a solution to the relaxed variational inequality. However, in order to show that a solution to (4.4) with $\frac{\mathrm {d}}{\mathrm {d}t} u \in L^{r^{*}}(I,X^{*})$ is likewise a solution to (4.3), additional assumptions are needed. Namely, it is required that in a decomposition of the functional *Φ*, associated with the decomposition of the monotone-convex functional as $\varphi(x,y) = \psi(y) - \psi(y) + \widehat {\varphi}(x,y)$, where $\psi: X \to \mathbb {R}$ is assumed to be convex and lower semi-continuous, the functional $\widehat {\varPhi }(u,\cdot)$ is continuous on *L* ^*r*^(*I*,*X*); for further details, see [8, Lemmas 2.2 and 2.6].

#### Lemma 4.1

([8, Lemmas 2.2 and 2.6])

*Under Hypothesis *4.1 *any solution to the integrated variational inequality *(4.3) *is a solution to the relaxed variational inequality *(4.4).

### A Uniqueness Result

#### A Uniqueness Result

The following result ensures uniqueness of the solution to the integrated variational inequality and thus to the evolutionary variational inequality, see also [8, Lemma 2.7]. Under the above stated additional requirements guaranteeing the equivalence of the integrated and relaxed formulations, also uniqueness of the solution to the relaxed variational inequality follows.

#### Lemma 4.2

([8, Lemma 2.7])

*Under Hypothesis *4.1 *there exists at most one solution to the integrated variational inequality *(4.3).

## Full Discretisations of Nonlinear Evolutionary Inequalities

In the following, we state the fully discrete counterparts of the considered nonlinear evolutionary variational inequality and of the integrated and relaxed formulations. Moreover, we deduce an existence and uniqueness result as well as a priori bounds for the discrete solution, which are an essential ingredient in the convergence analysis. Throughout, we assume that Hypothesis 4.1 on the governing nonlinear functional as well as Hypotheses 3.1 and 3.2 on the time and space discretisations are satisfied.

### Notations

For notational simplicity, as the integers $M,N \in \mathbb {N}$ corresponding to the time and space discretisations are meanwhile fixed, we do not indicate the dependence of the discrete solution values *U*_*m*−1,*i*_ and *u*_*m*−1_=*U*_*m*−1,*s*_ on *M*,*N*; as before, the initial condition and its approximation are distinguished by the notations *u*(0) and *u*_0_, respectively. We recall the compact vector notation *U*_*m*−1_=(*U*_*m*−1,1_,…,*U*_*m*−1,*s*_)^T^ and the relation *u*_*m*_=*U*_*m*−1,*s*_ for any 1≤*m*≤*M*. In view of the integrated formulation of the discrete variational inequality, we henceforth identify (in notation) the stage values and their piecewise constant interpolant *U*=(*U*_⋅,1_,…,*U*_⋅,*s*_)^T^, defined by

$$ U_{\cdot,i}(0) = u_0, \qquad U_{\cdot,i}(t) = U_{m-1,i}, \quad t \in(t_{m-1}, t_m],\ 1 \leq m \leq M, $$

for 1≤*i*≤*s*. Further, we employ the abbreviation *F*=(*F*_⋅,1_,…,*F*_⋅,*s*_)^T^ comprising (suitable) piecewise constant in time approximations *F*_⋅,*i*_ of the function $f \in L^{r^{*}}(I,X^{*})$ for 1≤*i*≤*s*. The space of -valued piecewise constant functions associated with an equidistant partition of the time interval is denoted by 
 see also Sect. 6.1.

### Fully Discrete Variational Inequality and Reformulations

#### Fully Discrete Nonlinear Evolutionary Variational Inequality

In regard to (3.7) we consider the following discrete analogue of Problem 4.1.

#### Problem 5.1

*For a given initial approximation* *u*_0_∈*K* ^(*N*)^ *and a given approximation* *F*∈(℘_0_(*I*,*X* ^∗^))^*s*^ *find* *U*∈(℘_0_(*I*,*K* ^(*N*)^))^*s*^ *such that* $U(0) = u_{0} \mathfrak {1}$ *and*

5.1 $$ \begin{aligned} & \biggl\langle \frac{1}{\tau } \widetilde {\mathfrak {A}}(U_{m-1} - u_{m-1} \mathfrak {1}) - \alpha F_{m-1} \bigg\vert V_{m-1} - U_{m-1} \biggr\rangle _{(X^{*})^s \times X^s} \\ &\quad{}+ \sum_{i = 1}^{s} \alpha_i \varphi (U_{m-1,i},V_{m-1,i} ) \geq0 \end{aligned} $$

*for all* *V*_*m*−1_∈(*K* ^(*N*)^)^*s*^ *and* 1≤*m*≤*M*.

#### Integrated Discrete Nonlinear Evolutionary Variational Inequality

For piecewise constant in time interpolants, the integral over the time domain simplifies to a Riemann sum. In particular, for any 1≤*i*≤*s* we have

$$ \varPhi (U_{\cdot,i}, V_{\cdot,i} ) = \int _{0}^{\mathrm{T}} \varphi \bigl(U_{\cdot,i}(t), V_{\cdot,i}(t) \bigr) \,\mathrm {d}t = \tau \sum _{m=1}^{M} \varphi (U_{m-1,i}, V_{m-1,i} ), $$

see also (4.1). As a consequence, multiplication of (5.1) by the time increment and summation yields the following discrete analogue of Problem 4.2.

#### Problem 5.2

*For* *u*_0_∈*K* ^(*N*)^ *and* *F*∈(℘_0_(*I*,*X* ^∗^))^*s*^ *find* *U*∈(℘_0_(*I*,*K* ^(*N*)^))^*s*^ *such that* $U(0) = u_{0} \mathfrak {1}$ *and*

5.2 $$ \begin{aligned} &\sum_{m=1}^{M} \bigl\langle \widetilde {\mathfrak {A}}(U_{m-1} - u_{m-1} \mathfrak {1}) - \tau \alpha F_{m-1} \big\vert V_{m-1} - U_{m-1} \bigr\rangle _{(X^{*})^s \times X^s} \\ &\quad{}+ \sum_{i = 1}^{s} \alpha_i \varPhi (U_{\cdot,i}, V_{\cdot,i} ) \geq0 \end{aligned} $$

*for all* *V*∈(℘_0_(*I*,*K* ^(*N*)^))^*s*^.

#### Relaxed Discrete Nonlinear Evolutionary Variational Inequality

Arguments similar to those used in the derivation of Lemma 4.1 yield a relaxed reformulation of the discrete nonlinear evolutionary variational inequality, see also (4.4) and [8, Lemmas 2.2 and 2.6]. Let *U*∈(℘_0_(*I*,*K* ^(*N*)^))^*s*^ with $U(0) = u_{0} \mathfrak {1}$ be a solution to (5.2), and assume *V*∈(℘_0_(*I*,*K* ^(*N*)^))^*s*^. Hypothesis 4.1 ensures that the functional *φ* and thus *Φ* is monotone; as a consequence, by (5.2) it follows with *V*_*m*−1_−*U*_*m*−1_ and associated values *v*_*m*−1_−*u*_*m*−1_ that

$$ \begin{aligned} &\sum_{i = 1}^{s} \alpha_i \varPhi (V_{\cdot,i}, U_{\cdot ,i} )\\ &\quad {}\leq- \sum_{i = 1}^{s} \alpha_i \varPhi (U_{\cdot,i}, V_{\cdot,i} ) \\ &\quad{}\leq\sum_{m=1}^{M} \bigl\langle \widetilde {\mathfrak {A}}(U_{m-1} - u_{m-1} \mathfrak {1}) - \tau \alpha F_{m-1} \big\vert V_{m-1} - U_{m-1} \bigr\rangle _{(X^{*})^s \times X^s} \\ &\quad{}= - \sum_{m=1}^{M} \bigl\langle \widetilde {\mathfrak {A}}\bigl(V_{m-1} - U_{m-1} - (v_{m-1} - u_{m-1}) \mathfrak {1}\bigr) \big\vert V_{m-1} - U_{m-1} \bigr\rangle _{(X^{*})^s \times X^s} \\ &\qquad{}- \sum_{m=1}^{M} \bigl\langle \widetilde {\mathfrak {A}}(V_{m-1} - v_{m-1} \mathfrak {1}) - \tau \alpha F_{m-1} \big\vert U_{m-1} - V_{m-1} \bigr\rangle _{(X^{*} )^s \times X^s}. \end{aligned} $$

A discrete analogue of the integration-by-parts formula relies on Lemma 3.1, and thus the telescopic identity

$$ \begin{aligned} &{-}\sum_{m=1}^{M} \bigl\langle \widetilde {\mathfrak {A}}\bigl(V_{m-1} - U_{m-1} - (v_{m-1} - u_{m-1}) \mathfrak {1}\bigr)\big\vert V_{m-1} - U_{m-1}\bigr\rangle _{(X^{*})^s \times X^s} \\ &\quad{}\leq\frac{1}{2} \sum_{m=1}^{M} \bigl(\|v_{m-1} - u_{m-1}\|_{H}^2 - \|v_{m} - u_{m}\|_{H}^2 \bigr) \\ &\quad{}= \frac{1}{2} \bigl(\|v_0 - u_0\|_{H}^2 - \|v_M - u_M\|_{H}^2 \bigr) \\ &\quad{}\leq\frac{1}{2} \|v_0 - u_0\|_{H}^2 \end{aligned} $$

implies the relaxed formulation given below.

#### Problem 5.3

*For* *u*_0_∈*K* ^(*N*)^ *and* *F*∈(℘_0_(*I*,*X* ^∗^))^*s*^ *find* *U*∈(℘_0_(*I*,*K* ^(*N*)^))^*s*^ *such that* $U(0) = u _{0} \mathfrak {1}$ *and*

5.3 $$ \begin{aligned} &\sum_{m=1}^{M} \bigl\langle \widetilde {\mathfrak {A}}(V_{m-1} - v_{m-1} \mathfrak {1}) - \tau \alpha F_{m-1}\big\vert U_{m-1} - V_{m-1}\bigr\rangle _{(X^{*})^s \times X^s} \\ &\quad{}+ \sum_{i = 1}^{s} \alpha_i \varPhi (V_{\cdot,i}, U_{\cdot,i} ) \leq\frac{1}{2} \|v_0 - u_0\|_{H}^2 \end{aligned} $$

*for all* *V*∈(℘_0_(*I*,*K* ^(*N*)^))^*s*^.

### Existence and Uniqueness of the Discrete Solution

#### Solvability of the Fully Discrete Inequality

The following theorem ensures the solvability of the fully discrete inequality and thus extends the result for the implicit Euler method and low-order finite element approximations given in [8, Lemma 3.5].

#### Theorem 5.1

*Under Hypotheses *3.1, 3.2 *and *4.1 *the existence and uniqueness of the discrete solution to *(5.1) *is ensured*.

#### Proof

For the following considerations it is convenient to rewrite (5.1) as

$$ \begin{aligned} & \langle \widetilde {\mathfrak {A}}U_{m-1} \vert V_{m-1} - U_{m-1} \rangle_{(X^{*})^s \times X^s} + \tau \sum_{i = 1}^{s} \alpha_i \varphi (U_{m-1,i}, V_{m-1,i} ) \\ &\quad{}\geq \langle \widetilde {\mathfrak {A}}u_{m-1} \mathfrak {1}+ \tau \alpha F_{m-1} \vert V_{m-1} - U_{m-1} \rangle_{(X^{*} )^s \times X^s}, \end{aligned} $$

where 1≤*m*≤*M*.

(a) Existence: The left-hand side in the above relation involves the positive definite matrix $\widetilde {\mathfrak {A}}$, the positive weights *α*_*i*_ for 1≤*i*≤*s* and the monotone-convex functional *φ*; the right-hand side is a fixed linear functional applied to the argument *V*_*m*−1_−*U*_*m*−1_. Thus, at each time step, the existence of the stage value *U*_*m*−1_ is ensured by [32, 39].

(b) Uniqueness: In order to prove uniqueness of the stage values, we assume the existence of two solutions *U*_*m*−1_ and $\widetilde {U}_{m-1}$ such that

$$ \begin{aligned} & \langle \widetilde {\mathfrak {A}}U_{m-1} \vert V_{m-1} - U_{m-1} \rangle_{(X^{*})^s \times X^s} + \tau \sum_{i = 1}^{s} \alpha_i \varphi (U_{m-1,i}, V_{m-1,i} ) \\ &\quad{}\geq \langle \widetilde {\mathfrak {A}}u_{m-1} \mathfrak {1}+ \tau \alpha F_{m-1} \vert V_{m-1} - U_{m-1} \rangle_{(X^{*} )^s \times X^s}, \\ & \langle \widetilde {\mathfrak {A}} \widetilde {U}_{m-1} \vert V_{m-1} - \widetilde {U}_{m-1} \rangle_{(X^{*})^s \times X^s} + \tau \sum _{i = 1}^{s} \alpha_i \varphi ( \widetilde {U}_{m-1,i}, V_{m-1,i} ) \\ &\quad{}\geq \langle \widetilde {\mathfrak {A}}u_{m-1} \mathfrak {1}+ \tau \alpha F_{m-1} \vert V_{m-1} - \widetilde {U}_{m-1} \rangle _{(X^{*})^s \times X^s}. \end{aligned} $$

Inserting $V_{m-1} = \widetilde {U}_{m-1}$ into the first relation and *V*_*m*−1_=*U*_*m*−1_ into the second identity, adding both relations and applying Lemma 3.1 yield

$$ \begin{aligned} \frac{1}{2} \|u_{m} - \widetilde {u}_{m}\|_{H}^2 &\leq \bigl\langle \widetilde {\mathfrak {A}}( U_{m-1} - \widetilde {U}_{m-1} ) \big\vert U_{m-1} - \widetilde {U}_{m-1} \bigr\rangle _{(X^{*})^s \times X^s} \\ &\leq \tau \sum_{i = 1}^{s} \alpha_i \bigl(\varphi (U_{m-1,i}, \widetilde {U}_{m-1,i} ) + \varphi ( \widetilde {U}_{m-1,i}, U_{m-1,i} ) \bigr) \leq0, \end{aligned} $$

due to the monotonicity of *φ* and the positivity of the weights *α*_*i*_>0 for 1≤*i*≤*s*, which in particular implies $u _{m} = \widetilde {u}_{m}$ for all 1≤*m*≤*M*. For the two-stage Radau IIA method the refined relation provided by Lemma 3.1 further yields $U_{m-1,1} = \widetilde {U}_{m-1,1}$ for 1≤*m*≤*M*; for a general stiffly accurate Runge–Kutta method, we instead make use of the fact that the matrix $\widetilde {\mathfrak {A}}$ is positive definite to conclude $U_{m-1} = \widetilde {U}_{m-1}$. □

### A Priori Estimates for the Discrete Solution

#### A Priori Estimates

A priori estimates for the discrete approximation values and increments are basic tools in the proof of the convergence result.

#### Theorem 5.2

*Under Hypotheses *3.1, 3.2 *and *4.1 *the solution to the discrete variational inequality *(5.1) *fulfills the a priori estimate*

$$ \|u_{M_0}\|_{H}^2 + \tau \sum _{m = 1}^{M_0} \| U_{m-1}\|_{X^s}^r \leq C(u_0,F) $$

*for any integer* 0<*M*_0_≤*M* *with constant*

$$ C = C(u_0,F) = C \Biggl(1 + \|u_{0}\|_{H}^2 + \tau \sum_{m = 1}^{M_0} \bigl\| F_{m-1}\bigr\| _{(X^{*})^s}^{r^{*}} \Biggr) $$

*depending in particular on the problem data*. *Furthermore*, *the estimate*

$$ \tau \sum_{m=1}^{M_0} \biggl\| \frac{1}{\tau } (U_{m-1} - u_{m-1} \mathfrak {1})\biggr\| _{X^{*}}^{r^{*}} \leq C(u_0,F) $$

*is valid*.

#### Proof

(a) Inserting *V*_*m*−1_=0 into the discrete variational inequality (5.1) yields

$$ \biggl\langle \frac{1}{\tau } \widetilde {\mathfrak {A}}(U_{m-1} - u_{m-1} \mathfrak {1}) - \alpha F_{m-1} \bigg\vert - U_{m-1} \biggr\rangle _{(X^{*})^s \times X^s} + \sum _{i = 1}^{s} \alpha_i \varphi ( U_{m-1,i},0 ) \geq0 $$

for 1≤*m*≤*M* and, by a slight reformulation the relation

$$ \begin{aligned} & \bigl\langle \widetilde {\mathfrak {A}}(U_{m-1} - u _{m-1} \mathfrak {1}) \big\vert U_{m-1} \bigr\rangle _{(X^{*})^s \times X^s} - \tau \sum_{i = 1}^{s} \alpha_i \varphi (U_{m-1,i},0 ) \\ &\quad{}\leq \tau \langle\alpha F_{m-1} \vert U_{m-1} \rangle_{(X^{*})^s \times X^s}. \end{aligned} $$

The coercivity of *φ* with exponent *r*∈[2,∞) implies

$$ C \tau \sum_{i = 1}^{s} \alpha_i \|U_{m-1,i}\|_{X}^r - C_0 \tau \sum_{i = 1}^{s} \alpha_i \leq- \tau \sum_{i = 1}^{s} \alpha_i \varphi (U_{m-1,i},0 ), $$

see also Hypothesis 4.1. By means of Lemma 3.1 and an application of Young’s inequality with additional (small) scaling *ε*>0 we obtain

$$ \begin{aligned} &\frac{1}{2} \bigl(\|u_{m}\|_{H}^2 - \|u_{m-1}\|_{H}^2 \bigr) + C \tau \sum_{i = 1}^{s} \alpha_i \|U_{m-1,i}\|_{X}^r - C_0 \tau \sum_{i = 1}^{s} \alpha_i \\ &\quad{}\leq \bigl\langle \widetilde {\mathfrak {A}}(U_{m-1} - u_{m-1} \mathfrak {1}) \big\vert U_{m-1} \bigr\rangle _{(X^{*})^s \times X^s} - \tau \sum _{i = 1}^{s} \alpha_i \varphi ( U_{m-1,i},0 ) \\ &\quad{}\leq \tau \|U_{m-1}\|_{X^s} \|\alpha F_{m-1}\|_{(X^{*})^s} \\ &\quad{}\leq C(r,\varepsilon) \tau \| U_{m-1}\|_{X^s}^r + C\biggl(r^{*}, \frac{1}{\varepsilon}\biggr) \tau \|\alpha F_{m-1}\|_{(X^{*})^s}^{r^{*}}, \end{aligned} $$

where $C(r,\varepsilon) = \frac{\varepsilon^{r}}{r}$ and $C(r^{*} ,\frac{1}{\varepsilon}) = \frac {1}{r^{*}} \frac{1}{\varepsilon^{r^{*}}}$, and thus by absorption, for *ε*>0 chosen sufficiently small, the relation

$$ \|u_{m}\|_{H}^2 - \|u_{m-1}\|_{H}^2 + \tau \|U_{m-1}\|_{X^s}^r \leq C \tau + C \tau \| F_{m-1}\|_{(X^{*})^s}^{r^{*}} $$

follows. For any positive integer 0<*M*_0_≤*M* corresponding to the time *T*_0_=*M*_0_ *τ* summation and a telescopic identity yield

$$ \|u_{M_0}\|_{H}^2 - \|u_{0}\|_{H}^2 + \tau \sum_{m = 1}^{M_0} \|U_{m-1}\|_{X^s}^r \leq C + C \tau \sum_{m = 1}^{M_0} \|F_{m-1}\|_{(X^{*})^s}^{r^{*}} $$

and thus the first bound results; the arising constant *C*>0 depends on the problem data and further on $\alpha, r, r^{*}, \varepsilon, \frac{1}{\varepsilon}$, the constants arising in the coercivity bound, and the final time *T*.

(b) Inserting *V*_*m*−1_=*U*_*m*−1_−*W*_*m*−1_∈(*K* ^(*N*)^)^*s*^ with ∥*W*_*m*−1,*i*_∥_*X*_=1 for any 1≤*i*≤*s* into (5.1) and using the growth condition provided by Hypothesis 4.1 yields the bound

$$ \begin{aligned} & \biggl\langle \frac{1}{\tau } \widetilde {\mathfrak {A}}(U_{m-1} - u_{m-1} \mathfrak {1}) \bigg\vert W_{m-1} \biggr\rangle _{(X^{*})^s \times X^s} \\ &\quad{}\leq \langle\alpha F_{m-1} \vert W_{m-1} \rangle_{(X^{*})^s \times X^s} + \sum_{i = 1}^{s} \alpha_i \varphi (U_{m-1,i}, U_{m-1,i} - W_{m-1,i} ) \\ &\quad{}\leq C \| F_{m-1}\|_{(X^{*})^s} \|W_{m-1}\|_{X^s} + C \sum _{i = 1}^{s} \alpha_i \bigl(1 + \|U_{m-1,i}\|_{X}^{r-1} \bigr) \|W_{m-1,i}\|_{X} \\ &\quad{}\leq C \bigl(1 + \|U_{m-1}\|_{X^s}^{r-1} + \|F_{m-1}\|_{(X^{*})^s} \bigr). \end{aligned} $$

Multiplication with the time increment and summation further implies

$$ \begin{aligned} &\tau \sum_{m=1}^{M_0} \biggl\| \frac{1}{\tau } \widetilde {\mathfrak {A}}(U_{m-1} - u_{m-1} \mathfrak {1})\biggr\| _{X^{*}}^{r^{*}} \\ &\quad{}\leq C T + C \tau \sum_{m=1}^{M_0} \|U_{m-1}\|_{X^s}^{r} + C \tau \sum_{m=1}^{M_0} \|F_{m-1}\|_{ (X^{*})^s}^{r^{*}} \end{aligned} $$

and thus, by the a priori bound for the stage values and the positive definiteness of the matrix $\widetilde {\mathfrak {A}}$ the stated result follows. □

#### Remark

In order to utilise the a priori bounds provided by Theorem 5.2 in the proof of the convergence result, it is essential to ensure that the arising quantities

$$ \|u_{0}\|_{H}^2,\quad \tau \sum _{m = 1}^{M} \|F_{m-1}\|_{(X^{*})^s}^{r^{*}}, $$

are bounded, independently of the integers $M,N \in \mathbb {N}$. Indeed, for the data *u*(0)∈*K* and *f*∈*L* ^*r*^(*I*,*X* ^∗^) a density result given in Sect. 6.2 will guarantee the existence of initial approximations $u_{0} = u^{(N)}_{0} \in K^{(N)}$ such that *u*_0_ converges to *u*(0) in *H* and of approximating functions  such that *F*_⋅,*i*_ converges to *f* in $L^{r^{*}}(I,X^{*})$ for 1≤*i*≤*s* as *M*,*N*→∞. As a consequence, for a general stiffly accurate Runge–Kutta method boundedness of the piecewise constant in time interpolant of the solution values (*u*_*m*_)_0≤*m*≤*M*_ in *L* ^∞^(*I*,*H*) as well as boundedness of the piecewise constant interpolant of the stage values (*U*_*m*−1_)_1≤*m*≤*M*_ in (*L* ^*r*^(*I*,*X*))^*s*^ is ensured. In addition, the refined bound for the two-stage Radau IIA method implies boundedness of the stages (*U*_*m*−1,1_)_1≤*m*≤*M*_ in the underlying Hilbert space *H*.

## Convergence Result

In the following, our concern is to establish a convergence result for the piecewise constant in time interpolant, under hypotheses close to the existence theory of nonlinear evolutionary equations and inequalities involving a monotone main part. Auxiliary definitions and results are given in Sects. 6.1 and 6.2. In order to keep the work at a reasonable length, especially in the proofs of the density and feasibility results, we only indicate the standard arguments used and instead refer to the literature for further details.

### Interpolation, Difference, and Restriction Operators

In this section, we collect several auxiliary results on the piecewise constant and linear in time interpolation operators as well as the difference and restriction operators associated with a stiffly accurate Runge–Kutta method.

In the following, we let  denote an arbitrary reflexive Banach space or a closed subset thereof. For notational simplicity, we omit the dependences of certain quantities on the integer $M \in \mathbb {N}$ as long as *M* is fixed; further, with a slight abuse of notation, we denote the vector of stage values by *Z*_*m*−1_ for 1≤*m*≤*M* and components of the associated piecewise constant interpolant by *Z*_*i*_ for 1≤*i*≤*s*. We recall that under Hypothesis 3.1 the nodes of the considered stiffly accurate Runge–Kutta method fulfill 0<*c*_*i*_<1 for 1≤*i*≤*s*−1 and *c*_*s*_=1, see also Sect. 3.1.1.

#### Piecewise Constant Interpolation Operator

The space of -valued piecewise constant functions associated with the partition of the time interval is given by 
 The piecewise constant interpolation operator associated with a stiffly accurate Runge–Kutta method  is defined by *Z*_*i*_(*t*)(0)=*z*(0) and *Z*_*i*_(*t*)=*z*(*t*_*m*−1_+*c*_*i*_ *τ*) for all *t*∈(*t*_*m*−1_,*t*_*m*_] and 1≤*m*≤*M*, where 1≤*i*≤*s*; in particular, it follows that *Z*_*s*_(*t*)=*z*(*t*_*m*_) for *t*∈(*t*_*m*−1_,*t*_*m*_] and 1≤*m*≤*M*. Moreover, for given discrete values  and  for 1≤*m*≤*M* the piecewise constant interpolants are given through *Z*_*i*_(0)=*z*_0_ and *Z*_*i*_(*t*)=*Z*_*m*−1,*i*_ for *t*∈(*t*_*m*−1_,*t*_*m*_] and 1≤*m*≤*M*, where 1≤*i*≤*s*. Throughout, we identify (in notation) the discrete values (*Z*_*m*−1_)_1≤*m*≤*M*_ and the associated piecewise constant interpolant ; in particular, we employ the abbreviation *z*=*Z*_*s*_.

#### Piecewise Linear Interpolation Operator

The space of -valued piecewise linear functions associated with the partition of the time interval is defined through 
 The piecewise linear interpolation operator  is given by $\widehat {z}(t) = z(t_{m-1}) + \frac{t - t_{m-1}}{\tau } (z(t_{m}) - z(t_{m-1}))$ for any *t*∈[*t*_*m*−1_,*t*_*m*_] and 1≤*m*≤*M*. Analogously, for discrete values  for 0≤*m*≤*M* the piecewise linear interpolant is defined by $\widehat {z}(t) = z_{m-1} + \frac{t - t_{m-1}}{\tau } (z_{m} - z_{m-1})$ for *t*∈[*t*_*m*−1_,*t*_*m*_] and 1≤*m*≤*M*.

#### Difference Operator

The finite difference operator associated with a stiffly accurate Runge–Kutta method  is defined through  for *t*∈(*t*_*m*−1_,*t*_*m*_] and 1≤*m*≤*M*, where we set *Z*=ℐ_0_(*z*). Analogously, for discrete values  and  for 1≤*m*≤*M* we consider the associated piecewise constant interpolant  and define  through 
 for any 1≤*m*≤*M*; as before, we denote *z*_*m*_=*Z*_*m*−1,*s*_ for 1≤*m*≤*M*.

#### Restriction (Integral Mean) Operator

The restriction operator associated with a stiffly accurate Runge–Kutta method  is defined through (ℳ*Z*)(0)=0 and 
 for *t*∈(*t*_*m*−1_,*t*_*m*_] and 1≤*m*≤*M*.

#### Remark

For the implicit Euler method we retain the defining relations 
 for *t*∈(*t*_*m*−1_,*t*_*m*_] and 1≤*m*≤*M*.

#### Basic Auxiliary Result

A discrete analogue of the integration-by-parts formula is provided by the following auxiliary result. We meanwhile assume that  defines a Hilbert space and consider the product space  with inner product and corresponding norm as defined in the introduction.

#### Lemma 6.1

*Provided that a stiffly accurate Runge–Kutta method satisfies Hypothesis *3.1, *the associated difference operator fulfills*

*for any*  *with* *Z*(0)=0.

#### Proof

It is straightforward to deduce the stated relation by applying the definition of the difference operator, Lemma 3.1 and the telescopic identity 
 where we recall the notation *z*_*m*_=*Z*_*m*−1,*s*_, and that *z*_0_=0 by assumption. □

#### Convergence Properties

Fundamental convergence properties of the interpolation, difference and restriction operators associated with a stiffly accurate Runge–Kutta method are collected in the following auxiliary result. For clarity, in the statement of the lemma we indicate the dependence of the operators  on the integer $M \in \mathbb {N}$; however, for notational simplicity, we omit the index in the proof of the result. We recall the abbreviation $\gamma^{-1} = \operatorname{diag} (\frac {1}{c_{1}},\dots,\frac{1}{c_{s}} )$.

#### Lemma 6.2

(i) *For any* 1≤*r*≤∞ *and*  *the piecewise constant and piecewise linear in time interpolants satisfy*

(ii) *For any* 1≤*r*<∞ *and*  *the restriction operator fulfills*

(iii) *Let*  *for some* 1≤*r*<∞. *Then*, *it follows that*

#### Proof

(i) It is straightforward to extend the following arguments specified for the case of a single component *s*=1 to the general case *s*≥1. By assumption, the function  is uniformly continuous, that is, for any *ε*>0 there exists $M \in \mathbb {N}$, sufficiently large, such that . As a consequence, for *r*=∞ we have 
 furthermore, for any 1≤*r*<∞ the claimed result follows: 



(ii) As before, it suffices to consider each component 1≤*i*≤*s* separately. The arguments are in the lines of [8] for the implicit Euler method.

(a) *r*=1: Employing a standard density argument, utilising the boundedness of ℳ, see Lemma 6.1, it suffices to consider a (left-continuous) step function . Choosing $M \in \mathbb {N}$ sufficiently large, we may assume that at a total number of *J* jumps, at most a single jump occurs on each subinterval [*t*_*m*−1_,*t*_*m*_]; we denote the left and right values of *z* at the subinterval by *z*_*m*−1,*ℓ*_ and *z*_*m*−1,*r*_, respectively. For instance, for the first subinterval [0,*τ*], we may suppose that a jump occurs at 0≤*ατ*≤*τ*. If *c*_*i*_≤*α* then 
 and otherwise, if *α*≤*c*_*i*_ then 
 where *t*∈[0,*τ*]. If *c*_*i*_≤*α* integration over the first subinterval yields 
 and otherwise, using that for $(1 + c_{i} - 2 \alpha) \frac{\alpha }{c_{i}}$ the maximum value $\frac{(1+c_{i})^{2}}{8 c_{i}} \leq\frac{1}{c_{i}}$ occurs at $\alpha= \frac{1+c_{i}}{4}$, we conclude 
 Due to the fact that any step function is of bounded variation, summation and taking the limit $\tau= \frac{T}{M} \to0$ as *M*→∞ yields the stated result 



(b) The extension to the case of exponents 1<*r*<∞ uses arguments detailed in [8, Proof of Lemma 3.3], which are not effected by the choice of the time discretisation method.

(iii) By the definition of the restriction and difference operators we obtain 
 for *t*∈(*t*_*m*−1_,*t*_*m*_] and 1≤*m*≤*M*. Thus, the claimed convergence follows by statement (ii). □

### A Density and Feasibility Result

The following density and feasibility results are utilised in the derivation of our convergence result for full discretisations of nonlinear evolutionary inequalities by stiffly accurate Runge–Kutta and *hp*-finite element methods.

#### A Density Result

Following the proof of [8] for the implicit Euler method, an analogous statement for the difference operator associated with a stiffly accurate Runge–Kutta method is obtained. We recall the definition of the solution space , see Sect. 4.1.

#### Lemma 6.3

*Suppose that*  *for some exponent* 2≤*r*<∞ *and that* $f \in L^{r^{*}}(I,X^{*})$ *for* $r^{*}= \frac{r}{r-1}$.

(i) *There exists a sequence* $(v^{(N)}_{0})_{N \in \mathbb {N}}$ *such that* $v^{(N)}_{0} \in K^{(N)}$ *for* $N \in \mathbb {N}$ *and*
  $$ v^{(N)}_{0} \overset{N \to\infty}{\longrightarrow} v(0) \quad\textit{in } H. $$
(ii) *There exists a sequence of positive integer numbers* $(M_{N})_{N \in \mathbb {N}}$ *with corresponding subsequence of equidistant partitions* $(\mathbb {I}^{(M_{N})} )_{M_{N}\in \mathbb {N}}$ *and a sequence* $(v^{(N)})_{N \in \mathbb {N}}$ *with*  *for* $N \in \mathbb {N}$ *such that*

(iii) *There exists a sequence* $(F^{(N)})_{N \in \mathbb {N}}$ *such that*  *for all* $N \in \mathbb {N}$ *and*
  $$ F^{(N)}\overset{N \to\infty}{\longrightarrow} f \quad\textit{in } L^{r^{*}}\bigl(I,X^{*}\bigr). $$

#### Proof

For a given function  we consider the piecewise constant and linear in time interpolants  for *ℓ*=0,1. An application of Lemma 6.2 ensures strong convergence of the interpolants 
 as well as convergence of the finite differences 
 The approach used in [8] is to construct piecewise constant and linear in time approximations to the discrete values $(v(t_{m}))_{m=1}^{M}$. Differentiation of the piecewise linear approximation yields an approximation to the first time derivative $\frac{\mathrm {d}}{\mathrm {d}t} v$. Furthermore, approximations of the initial value *v*(0) in *H* and of the function *f* in $L^{r^{*}}(I,X^{*})$ are constructed. For detailed explanations, we refer to [8, Lemma 3.7]. □

#### A Feasibility Result

The following result ensures that the considered time and space approximations admit limit points that belong to the convex set *K*. The proof of the lemma is found in [8, Proof of Lemma 3.13] and [20], see also Sect. 3.2.2 for the definition of *G*-convergence and Sect. 4.1 for the definition of *L* ^*r*^(*I*,*K*).

#### Lemma 6.4

([8, Lemma 3.13])

*Assume that Hypotheses *3.1, 3.2 *and *4.1 *are satisfied*. *Suppose that the stages* $U_{m-1} = U^{(M,N)}_{m-1} \in(K^{(N)})^{s}$ *for* 1≤*m*≤*M* *with associated piecewise constant and piecewise linear interpolants* $U^{(M,N)}, \widehat {U}^{(M,N)}$ *are solutions to Problem *5.1. *Assume further that the sequence of subsets* $(K^{(N)})_{N \in \mathbb {N}}$ *G*-*converges to K* *for* *N*→∞. *Then*, *any limit point of U* ^(*M*,*N*)^ *or* $\widehat {U}^{(M,N)}$, *respectively*, *with respect to weak convergence in* (*L* ^*r*^(*I*,*X*))^*s*^ *belongs to *(*L* ^*r*^(*I*,*K*))^*s*^.

### Main Result

#### Convergence Result

With the above stated auxiliary results at hand, we are able to establish the following convergence result for full discretisations of Problem 4.1 by stiffly accurate Runge–Kutta methods and *hp*-finite element approximations. In regard to the length of the work, we focus on the relaxed formulation of the problem; in this case, differentiability in time is not needed and thus it suffices to employ the first a priori estimate for the discrete solution.

#### Theorem 6.5

*Let* *u*(0)∈*K* *and* *f*∈*L* ^*r*^(*I*,*X* ^∗^). *Assume that the nonlinear functional φ* *defining Problem *4.1 *and the related functional Φ* *fulfill Hypotheses *4.1 *and *4.2. *Suppose that the stiffly accurate Runge–Kutta and* *hp*-*finite element time and space discretisations satisfy Hypothesis *3.1 *as well as Hypothesis *3.2 *and that the sequence* $(K^{(N)} )_{N \in \mathbb {N}}$ *G*-*converges to K* *for* *N*→∞. *Assume further that the initial approximations* $u^{(M,N)}_{0} = u^{(N)}_{0} \in K^{(N)}$ *such that* $u^{(N)}_{0}$ *converges to u*(0) *in H* *and the approximations*  *such that* $F^{(M,N)}_{\cdot,i}$ *converges to f* *in* $L^{r^{*}}(I,X^{*})$ *for* 1≤*i*≤*s* *as* *M*,*N*→∞ *are chosen in accordance with Lemma *6.3. *Then*, *the following statement is valid for the solution* $(U^{(M,N)} _{m-1})_{m=1}^{M} \subset K^{(N)}$ *to Problem *5.1 *and the associated piecewise constant interpolant* .

*The sequence* $(U^{(M,N)})_{M,N \in \mathbb {N}}$ *possesses limit points with respect to weak convergence in *(*L* ^*r*^(*I*,*X*))^*s*^, *and the associated sequence* $(u^{(M,N)})_{M,N \in \mathbb {N}}$ *given by* $u^{(M,N)}= U^{(M,N)}_{\cdot,s}$ possesses limit points with respect to weak* *convergence in L* ^∞^(*I*,*H*). *Any limit point of* $(U^{(M,N)})_{M,N \in \mathbb {N}}$ *belongs to *(*L* ^*r*^(*I*,*X*))^*s*^ *and is a solution to Problem *4.3, *the relaxed formulation of the evolutionary inequality*.

#### Proof

For simplicity, we write $\tau = \tau^{(M)}= \frac{T}{M}$ as well as $t_{m} = t^{(M)}_{m} = m \frac{T}{M}$, where 0≤*m*≤*M*, for short.

The existence of limit points with the stated properties follows from the a priori estimate for the solution and stage values, respectively, provided by Theorem 5.2, that is, from the boundedness of the associated piecewise constant interpolants in *L* ^∞^(*I*,*H*) and (*L* ^*r*^(*I*,*X*))^*s*^, respectively. By the feasibility result, Lemma 6.4, any limit point *U* of *U* ^(*M*,*N*)^ belongs to (*L* ^*r*^(*I*,*K*))^*s*^. For an element , in accordance with the density result, we choose a common subsequence of integer pairs (*M*_*N*_,*N*) and sequences of elements $v^{(N)}_{0} \in K^{(N)}$ as well as  such that 
 see Lemma 6.3; here, for simplicity, we do not distinguish the sequence of pairs (*M*_*N*_,*N*) and the chosen subsequence in notation, and we set $U^{(N)} _{m-1} = U^{(M,N)}_{m-1}$ etc. Inserting the piecewise constant interpolant  defined by $V^{(N)}_{m-1} = (v^{(N)}(t_{m-1} + c_{1} \tau ), \dots, v^{(N)}(t_{m-1} + c_{s} \tau ))^{\mathrm{T}}$ for 1≤*m*≤*M* into the relaxed formulation of the discrete variational inequality (5.3) yields

$$ \begin{aligned} &\tau \sum_{m=1}^{M} \biggl\langle \frac {1}{\tau } \widetilde {\mathfrak {A}}\bigl(V^{(N)}_{m-1} - v^{(N)}_{m-1} \mathfrak {1}\bigr) - \alpha F^{(N)}_{m-1}\bigg\vert U^{(N)}_{m-1} - V^{(N)}_{m-1}\biggr\rangle _{(X^{*})^s \times X^s} \\ &\quad{}+ \sum_{i = 1}^{s} \alpha_i \varPhi \bigl(V^{(N)}_{\cdot,i}, U^{(N)} _{\cdot ,i} \bigr) \leq\frac{1}{2} \bigl\| v^{(N)}_0 - u^{(N)}_0\bigr\| _{H}^2. \end{aligned} $$

Performing the limit *N*→∞, using strong convergence of $u^{(N)} _{0}$ to *u*_0_ in *H*, of $v^{(N)}_{0}$ to *v*(0) in *H*, of *F* ^(*N*)^ to $f \mathfrak {1}$ in (*L* ^*r*^(*I*,*X* ^∗^))^*s*^, of  to $\alpha \mathfrak {1}\frac{\mathrm {d}}{\mathrm {d}t} v$ in $(L^{r^{*}}(I,X^{*}))^{s}$, weak convergence of *U* ^(*N*)^−*V* ^(*N*)^ to $(u-v) \mathfrak {1}$ in (*L* ^*r*^(*I*,*X*)) and the required LSC condition, see Hypothesis 4.2, imply that *u* solves the relaxed formulation of the variational inequality

$$ \int_{0}^{\mathrm{T}} \biggl\langle \frac{\mathrm {d}}{\mathrm {d}t} v(t) - f(t) \bigg\vert u(t) - v(t) \biggr\rangle _{X^{*}\times X} \,\mathrm {d}t + \varPhi (v, u) \leq\frac{1}{2} \bigl\| v(0) - u_0\bigr\| _{H}^2, $$

see (4.4); we recall that by the consistency condition of Hypothesis 3.1 we have $\mathfrak {A} \mathfrak {1}= \mathfrak {c}$ and consequently $\widetilde {\mathfrak {A}}\gamma \mathfrak {1}= \alpha \mathfrak {A}^{-1} \mathfrak {c}= \alpha \mathfrak {1}$ as well as *α*_1_+⋯+*α*_*s*_=1. □

## Conclusions

In the present work, our main objective has been to provide a convergence analysis for full discretisations by stiffly accurate Runge–Kutta methods and *hp*-finite element approximations, applied to nonlinear evolutionary inequalities stemming for instance from a Signorini-type initial-boundary value problem. Following [8] it is straightforward to extend the analysis to time-dependent monotone-convex functionals involving an explicit time-dependency. Further related questions that remain open comprise the extension of the convergence result to nonuniform time grids and other time integration methods such as BDF-methods. In this work, we studied the relaxed formulation of the problem, and it remains open to extend the arguments to the (integrated) variational inequality. For future work, it is also intended to numerically exploit the attainable convergence rate and to deduce error estimates for problems, where the solution satisfies certain regularity requirements; in this context, we refer to Hölder regularity results in space and time for parabolic domain obstacle problems in [31] and to *L* ^2^(*I*,*L* ^*r*^(*Ω*)) regularity of *∂*_*t*_∇*u* for the solution *u* to an evolutionary inequality involving the *r*-Laplacian in [30]. However, even for free problems on the boundary like a Signorini-type initial-boundary value problem with regular data it is supposed that due to the imposed constraints a severe order reduction compared to classic problems will occur, in general, and that a higher-order convergence rate is only obtained in the interior of the spatial domain.
